# Fe/S Redox-Coupled Mercury Transformation Mediated by *Acidithiobacillus ferrooxidans* ATCC 23270 under Aerobic and/or Anaerobic Conditions

**DOI:** 10.3390/microorganisms11041028

**Published:** 2023-04-14

**Authors:** Yue Liu, Chenyun Gu, Hongchang Liu, Yuhang Zhou, Zhenyuan Nie, Yirong Wang, Lu Chen, Jinlan Xia

**Affiliations:** 1 School of Minerals Processing and Bioengineering, Central South University, Changsha 410083, China; 2 Key Lab of Biometallurgy of Ministry of Education of China, Central South University, Changsha 410083, China

**Keywords:** acid mine drainage, acidophiles, transcriptome analysis, Fe/S redox, mercury transformation

## Abstract

Bioleaching processes or microbially mediated iron/sulfur redox processes in acid mine drainage (AMD) result in mineral dissolution and transformation, the release of mercury and other heavy metal ions, and changes in the occurrence forms and concentration of mercury. However, pertinent studies on these processes are scarce. Therefore, in this work, the Fe/S redox-coupled mercury transformation mediated by *Acidithiobacillus ferrooxidans* ATCC 23270 under aerobic and/or anaerobic conditions was studied by combining analyses of solution behavior (pH, redox potential, and Fe/S/Hg ion concentrations), the surface morphology and elemental composition of the solid substrate residue, the Fe/S/Hg speciation transformation, and bacterial transcriptomics. It was found that: (1) the presence of Hg^2+^ significantly inhibited the apparent iron/sulfur redox process; (2) the addition of Hg^2+^ caused a significant change in the composition of bacterial surface compounds and elements such as C, N, S, and Fe; (3) Hg mainly occurred in the form of Hg^0^, HgS, and HgSO_4_ in the solid substrate residues; and (4) the expression of mercury-resistant genes was higher in earlier stages of growth than in the later stages of growth. The results indicate that the addition of Hg^2+^ significantly affected the iron/sulfur redox process mediated by *A. ferrooxidans* ATCC 23270 under aerobic, anaerobic, and coupled aerobic–anaerobic conditions, which further promoted Hg transformation. This work is of great significance for the treatment and remediation of mercury pollution in heavy metal-polluted areas.

## 1. Introduction

Heavy metal pollution is a major environmental problem in the world today, and continued heavy metal pollution poses a potentially significant threat to almost all forms of life in the environment [1,2]. Mercury is a pollutant with high toxicity, persistence, enrichment, and mobility that can exist in inorganic forms, such as divalent mercury ions (Hg^2+^) and elemental mercury (Hg^0^), and can be transformed in situ to methylated forms. The toxicity of mercury is determined by its speciation and its occurrence forms [3,4,5]. The microbially mediated dissolution of sulfur-containing minerals is an important source of mercury pollution in mining areas [6].

Mining areas are an important site of mercury release and migration and pose a major threat to the surrounding soil and water environment. During the occurrence and development of mercury pollution, extremophilic microorganisms in the mining areas (such as extremely acidophilic, thermophilic, salt-tolerant, and heavy metal ion-tolerant microorganisms) occur in the main niche [7,8], which promotes the interaction between minerals, microorganisms, and metals; leads to the further dissolution and transformation of minerals; and significantly changes the occurrence forms and toxicity of mercury [9,10]. In the acid mine drainage environment, chemoautotrophic acidophilic iron- and sulfur-oxidizing microorganisms can oxidize and leach sulfide ores, and chemoautotrophic microorganisms metabolize organic carbon to produce organic acids and promote the dissolution of secondary oxidized minerals, which can lead to the release of mercury into water and soil, resulting in mercury pollution [11].

*Acidithiobacillus ferrooxidans*, a typical chemoautotrophic Gram-negative bacterium, can survive and reproduce in the acid mine drainage (AMD) of sulfide mineral mining environments. Under aerobic conditions, *A. ferrooxidans* can oxidize Fe^2+^ and S^0^ and reduce sulfur compounds to obtain energy [12,13]. At the same time, *A. ferrooxidans* can also obtain energy under anaerobic conditions with S^0^, H_2_, and Fe^2+^ as electron donors and Fe^3+^ and S^0^ as electron acceptors [14,15]. Through a genomic study of *A. ferrooxidans*, it was found that it has resistance to mercury and other heavy metals [16]. As early as 1982, it was found that the resistance mechanism of *A. ferrooxidans* to mercury mainly depends on mercury reduction by *merA* encoded by the *mer* operon [17,18]. In addition to the mercury reduction mechanism involved in the *mer* operon, cytochrome *c* oxidase also plays an important role in mercury reduction [18]. However, there are few studies on the combination of iron and sulfur metabolism in *A. ferrooxidans* and its mercury transformation mechanism. Additionally, the relationship between iron/sulfur speciation transformation and mercury fate has rarely been studied.

Thus, in the present study, by changing the oxygen state in the environment, the coupling relationship between iron and sulfur redox and mercury transformation behavior under aerobic, anaerobic, and aerobic–anaerobic coupling conditions was revealed, and the mercury transformation mechanism mediated by iron and sulfur redox of *A. ferrooxidans* was expounded. This study lays a foundation for further understanding the role and regulatory mechanism of extreme acidophilic microorganisms in the geochemical cycle of mercury.

## 2. Materials and Methods

### 2.1. Strain and Culture Conditions

The strain *A. ferrooxidans* ATCC 23270 (accession number of 16S rRNA in GenBank: AB189135) used in this study was preserved at the Key Laboratory of Biometallurgy of the Ministry of Education of China, Changsha, China. The basic medium for cultivation of this strain was 9K basic medium, which includes 3.0 g/L (NH_4_)_2_SO_4_, 0.1 g/L KCl, 0.5 g/L K_2_HPO_4_, 0.5 g/L MgSO_4_·7H_2_O, and 0.01 g/L Ca(NO_3_)_2_. Before the experiment, the strain was acclimated with 44.7 g/L FeSO_4_·7H_2_O, 10.0 g/L S^0^, and 10.0 g/L pyrite, respectively, as energy substrates to make it grow stably under each cultivation condition.

### 2.2. Mineral Samples

The original pyrite was provided by the Key Laboratory of Biometallurgy of Ministry of Education of China, Changsha, China. The analytical elemental sulfur was purchased from Sigma-Aldrich (St. Louis, MO, USA). The X-ray diffraction (XRD) analysis showed that the phase compositions of the pristine S^0^ and pyrite were basically pure (Figure S1). The pristine S^0^ and pyrite were ground to a fine powder and then passed through a 200–400 mesh screen with particle sizes of 38–75 μm.

### 2.3. Experiments

To study the mechanism of the iron and sulfur redox-coupled mercury transformation of *Acidithiobacillus ferrooxidans* ATCC 23270, four different cultivation conditions for *A. ferrooxidans* ATCC 23270 were designed: (i) the aerobic Fe^2+^ oxidation group, (ii) the aerobic S^0^ oxidation group, (iii) the anaerobic Fe^3+^ reduction coupled with S^0^ oxidation group, and (iv) the aerobic–anaerobic coupling group. All groups were cultured in triplicate with 1 mg/L [Hg^2+^]- Hg(NO_3_)_2_ and without Hg. During the experiment, the mass of the conical flask was weighed every day, and the liquid lost due to evaporation was supplemented with sterile deionized water. The initial inoculated cell density was 8 × 10^7^ cells/mL. The initial pH of the medium was adjusted with 2 M sulfuric acid to pH 2.0. The sterile experiment with 1 mg/L [Hg^2+^] was used as the sterile control group.

For (i) the aerobic Fe^2+^ oxidation and (ii) the aerobic S^0^ oxidation groups, the experiments were carried out in a 250 mL conical flask containing 100 mL sterilized basic medium and 1.00 g S^0^ or 4.47 g FeSO_4_·7H_2_O. The inoculated conical flask was placed in an oscillating incubator (ZQZY-A8) and cultured at a constant temperature of 30 °C and a rotational speed of 180 rpm. For (iii) the anaerobic Fe^3+^ reduction coupled with S^0^ oxidation group, the experiments were carried out in a 250 mL conical flask containing 100 mL sterilized basic medium with 1.00 g S^0^ and 0.20 g Fe_2_(SO_4_)_3_. The inoculated conical flask was placed into an anaerobic incubator (LAI-3T-N1) and cultured at a constant temperature of 30 °C. For (iv) the aerobic–anaerobic coupling group, the experiments were carried out in a 250 mL conical flask containing 100 mL sterilized basic medium with 1.00 g FeS_2_. Firstly, the inoculated conical flask was placed in an oscillating incubator (ZQZY-A8) and cultured at a constant temperature of 30 °C and a rotating speed of 180 rpm under aerobic conditions. When the pH of the system was at the lowest and stable for 2–3 days, the system was transferred to an anaerobic incubator (LAI-3T-N1) and cultured at a constant temperature of 30 °C. During the experiment, the mass of the conical flask was weighed every day, and the liquid lost due to evaporation was supplemented with sterile deionized water.

### 2.4. Analytical Methods

Solution samples were collected at regular intervals to detect pH, ORP, cell concentration, soluble Fe^3+^ concentration, and total Fe/S/Hg concentration. The pH and ORP values of the solution were measured with a pH meter (PHS-3C) and a platinum electrode with a Hg/HgCl_2_ reference electrode, respectively. The cell density was directly counted using light microscopy (Nexcopy NE900) with a blood corpuscle counter (XB-K-25). The ferric ion concentration ([Fe^3+^]) and total iron concentration ([Total Fe]) were determined using the sulfosalicylic acid method, and the ferric ion concentration ([Fe^2+^]) was obtained as [Total Fe]-[Fe^3+^]. The total sulfur concentration ([SO_4_^2−^]) and total mercury concentration ([Hg^2+^]) in the solution were determined using ICP-OES (SPECTROBLUE, FMX26).

Transmission electron microscopy (TEM) (JEM-1230) was used to observe the sliced bacterial cells deposited on copper wire. A TEM energy-dispersing X-ray spectrometer (EDX) (FEI/Talos F200i G2) was used to analyze the distribution of elements in the cells. Scanning electron microscopy–energy dispersive spectroscopy (SEM-EDS) (MIRA 3 LMU) was used to analyze the cell surface morphology and element distribution.

The surface morphology and element distribution of the substrate residue were characterized with SEM-EDS (MIRA 3 LMU). The phase composition of the substrate residue was characterized using Fourier-transform infrared spectroscopy (FT-IR) (SpectrumTwo), Raman spectroscopy (Renishaw InVia Qontor), and X-ray diffraction (XRD). The scanning angle of XRD analysis ranges from 5° to 80°, and the scanning speed is 2°/min.

The morphology transformation of Fe, S, and Hg elements in the substrate residue was characterized using X-ray photoelectron spectroscopy (XPS) and synchrotron radiation X-ray absorption near edge structure spectroscopy (XANES). XPS spectra of substrate residue samples were collected using Thermo Scientific ESCALAB Xi+ photoelectron spectroscopy. The linear component fitting of samples was performed in CasaXPS software. The electron binding energy (BE) was corrected with the absorption peak of C 1*s* 284.8 eV. The XANES spectra of the S K-edge in the substrate residue samples were collected using the X-ray 4B7A beamline at the synchrotron radiation facility of the Beijing Institute of High Energy Physics, Chinese Academy of Sciences. The normalization and linear fitting of sample spectra were carried out in Athena software in the IFEFFIT program [19].

### 2.5. Microbial Transcriptome Sequencing Analysis

During the experiment, a whole bottle of culture system was taken out from each parallel group experiment with bacteria, and the substrate residue was removed with low-speed centrifugation. The supernatant was taken for high-speed centrifugation to obtain bacterial cells, washed with phosphate-buffered saline (PBS) buffer at pH 7.0 2–3 times, quickly frozen in liquid nitrogen after centrifugation, and stored at −80 °C. The TRIzol method was used to extract total RNA from the sample, and a Thermo NanoDrop One and Agilent 4200 Tape Station were used for sample inspection. After the inspection, the results were qualified, and the Epicenter Ribo-Zero rRNA Removal Kit was used to remove ribosomal RNA. The cDNA Library was constructed using the NEBNextő Ultra II Directional RNA Library Prep Kit for Illumina, and PE150 was sequenced using the Illumina high-throughput sequencing platform. Quality inspection, library construction, and sequencing were all undertaken by Guangdong Mega Gene Technology Co., Ltd.

Gene expression was quantified with RSEM software [20], and differential gene expression was analyzed in DESeq2 software after read counts were obtained [21]. After comparing the sequenced genes with the GO databases [22,23], the differentially expressed genes were analyzed and annotated for gene functional classification. A paired *t*-test with a *p*-value cutoff of 0.05 was then used to analyze statistically significant changes in the gene expression in the normalized data. We manually classified statistically significant genes whose gene expression changed by more than twofold according to function. After completing the functional annotation, ClusterProfiler software [24] was used for GO enrichment analysis.

## 3. Results and Discussion

### 3.1. Bacterial Growth and Fe/S Oxidation

**Figure 1:**
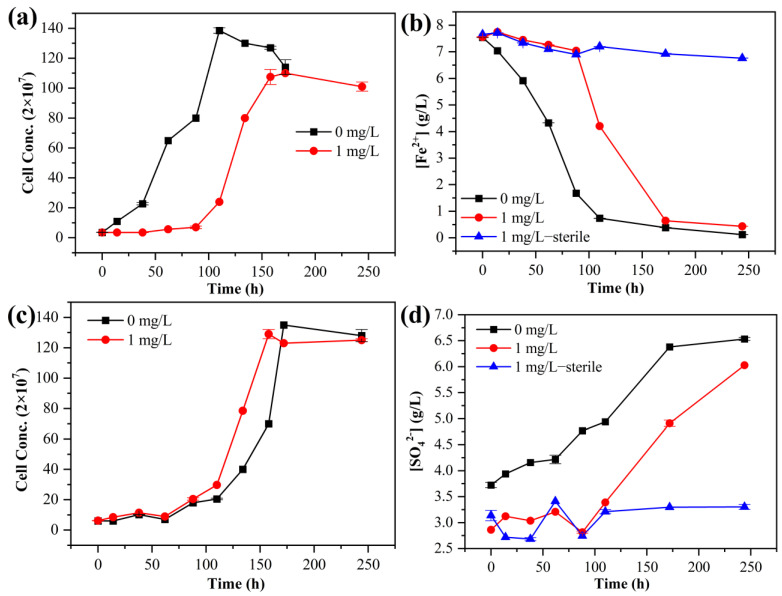

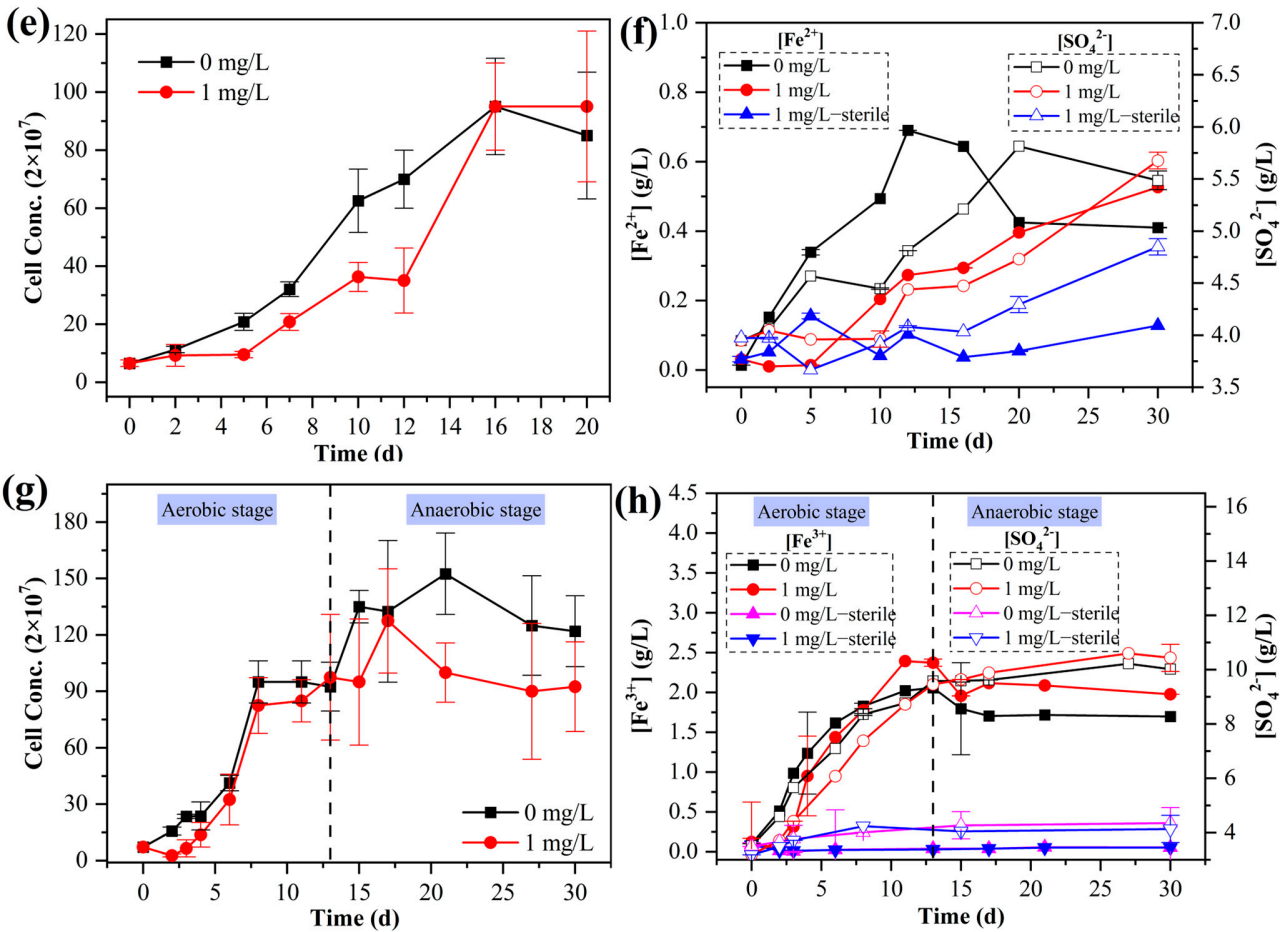
Bacterial growth and Fe/S oxidation for the groups of *A. ferrooxidans* ATCC 23270 grown with Fe^2+^ (**a**,**b**), S^0^ (**c**,**d**), anaerobic Fe^3+^ reduction coupled with S^0^ oxidation (**e**,**f**), and aerobic–anaerobic coupling (**g**,**h**) with and without the addition of 1 mg/L Hg^2+^.

Figure 1 shows the curves of cell density and Fe/S oxidation over time for all experimental groups with 0 and 1 mg/L [Hg^2+^]. Under the condition of aerobic Fe^2+^ oxidation, the group with Hg^2+^ showed an obvious lag phase at 0–88 h, in which the bacterial density did not increase significantly, then it entered the logarithmic phase at 88–158 h, and the bacterial growth reached the stationary phase at 158 h. In contrast, the group without Hg^2+^ had no obvious lag phase, and the bacteria entered the logarithmic phase within 10 h and reached the stationary phase after 88 h (Figure 1a). [Total Fe] and [Fe^2+^] showed a decreasing trend in all systems (Figure 1b and Figure S2a). [Total Fe] and [Fe^2+^] for the group without Hg^2+^ decreased rapidly without a significant delay and finally reached the lowest concentration of 0.68 mg/L. However, there were no significant changes in [Total Fe] or [Fe^2+^] within 0–88 h in the group of bacteria with Hg^2+^, and they then rapidly decreased after 88 h. Compared with the group without Hg^2+^, the decreases in [Total Fe] and [Fe^2+^] in the solution of the group with Hg^2+^ were slower. These results indicate that the bacterial growth was inhibited in the presence of 1 mg/L Hg^2+^ due to the toxic effect of Hg^2+^, with the inhibition of microbial Fe^2+^ oxidation at the early phase. Furthermore, in addition to the oxidation of *A. ferrooxidans* ATCC 23270, oxygen in the air also contributes to a small degree to the oxidation of Fe^2+^ in the solution. When Fe^2+^ is oxidized to produce a large amount of Fe^3+^, jarosite will be formed under the appropriate pH value, resulting in a decrease in the total Fe concentration in the solution.

Under aerobic S^0^ oxidation, the bacterial density of all systems showed a trend of rising at the initial stage and then changing a little (Figure 1c). There was no significant change in 0–62 h, indicating that the bacterial growth was in the lag phase. Compared with the group without Hg^2+^, the group with Hg^2+^ showed slower bacterial growth and a longer time to reach the stationary phase. The presence of Hg^2+^ has a strong stress effect on the early stage of microbial growth, leading to the extension of the lag phase. With the adaptation of the mercury-resistant mechanism and the gradual release of mercury stress, *A. ferrooxidans* ATCC 23270 gradually enters the logarithmic phase. There was no significant change in [SO_4_^2−^] for the sterile control group, while [SO_4_^2−^] for all bio groups (with *A. ferrooxidans* ATCC 23270) showed an increasing trend and increased with bacterial growth (Figure 1d). [SO_4_^2−^] had no significant change before 88 h in the bio group with Hg^2+^. After 88 h, it began to increase rapidly, and the inhibition was relieved to a certain extent. However, compared with the control group without Hg^2+^, the increase in [SO_4_^2−^] was slower over time. This result shows that *A. ferrooxidans* ATCC 23270 significantly inhibits the oxidation process of elemental sulfur to produce acid, and the presence of Hg^2+^ has a certain negative effect on *A. ferrooxidans* ATCC 23270 sulfur metabolism.

Under the conditions of anaerobic Fe^3+^ reduction coupled with S^0^ oxidation, the bacterial density in the solution showed an overall increasing trend (Figure 1e). There was no obvious lag phase for the group without Hg^2+^, and the bacterial concentration began to enter the logarithmic phase at day 2. On the other hand, bacteria in the experimental group supplemented with 1 mg/L [Hg^2+^] were in the lag phase at 0–5 days, and their growth was significantly inhibited, which may have been due to the stress effect of Hg^2+^ leading to the low activity of bacteria. The concentration of bacteria increased significantly after 5 days. [Fe^2+^] in the solution of the bacterial system showed an increasing trend (Figure 1f), while [Total Fe] had no significant change (Figure S2b). In the control group without Hg^2+^, [Fe^2+^] increased rapidly immediately after inoculation, indicating that the bacteria grew well with the high activity of the anaerobic Fe^3+^ reduction coupled with S^0^ oxidation (Figure 1f). However, the bacteria under the influence of Hg^2+^ showed a weaker Fe^2+^ reduction ability and slower [Fe^2+^] increase compared with the group without Hg^2+^. There was no significant change in the Fe^2+^ concentration in the aseptic system. The concentration changes of different forms of Fe in solution indicate that *A. ferrooxidans* ATCC 23270 in the system reduces Fe^3+^. The reason why there was no significant change in [Total Fe] may be that most Fe existed in the solution during bacterial action and did not migrate to the substrate residue and cell surface. Meanwhile, [SO_4_^2−^] for the bio group showed an increasing trend, while [SO_4_^2−^] for the sterile group showed no significant change (Figure 1f). In the bacterial system without Hg^2+^, [SO_4_^2−^] increased rapidly due to the oxidation of bacteria and reached the highest level at day 20. However, [SO_4_^2−^] for the bio group in the presence of Hg^2+^ showed an increasing trend from day 10, and the increase was slower than that for the bio group without Hg^2+^.

Under the conditions of the aerobic–anaerobic coupling experiment (Figure 1g), the bacterial density showed an increasing trend under aerobic conditions and reached a stable concentration at day 8. After entering the anaerobic environment, the concentration of bacteria increased again and then decreased and reached stability at day 21. This was because, during the aerobic culture stage, the addition of Hg^2+^ inhibited the initial growth of bacteria. With the progress in the mercury conversion process, the stress effect of Hg^2+^ was almost lifted at day 11. Therefore, after entering the anaerobic culture stage, the growth process of bacteria was hardly inhibited by Hg^2+^. Under aerobic conditions, the oxidation of pyrite by bacteria resulted in an increase in [Fe^3+^] in solution (Figure 1h), and the [Total Fe] also showed an increasing trend (Figure S2c). After entering the anaerobic stage, the iron reduction of bacteria resulted in a decrease in [Fe^3+^] in the solution, but [Total Fe] did not change significantly. Since most Fe in the solution exists in the form of Fe^3+^, when Fe^3+^ is reduced by bacteria under anaerobic conditions, it exists in the form of Fe^2+^ in the solution. As a result, [Total Fe] shows no obvious change when the bacterial growth environment is anaerobic. There was no significant change in [Fe^3+^] in the sterile system, but [Total Fe] increased slightly, which may have been caused by the dissolution of pyrite in the medium. In addition, compared with the control group without Hg^2+^, the increase in [Fe^3+^] in the solution of the bacterial system with Hg^2+^ was slower with time, but [Fe^3+^] was higher after day 11. This result indicates that the presence of Hg^2+^ inhibited the activity of bacterial growth at the initial stage, and the metabolic activity of bacterial growth was improved after the removal of the inhibition effect. [SO_4_^2−^] in the solution of all experimental systems showed an increasing trend due to the leaching effect of pyrite by bacteria and the dissolution effect of pyrite itself at low pH values (Figure 1h). The addition of Hg^2+^ has an inhibitory effect on the growth and metabolism of bacteria, resulting in the slow elevation of solution [SO_4_^2−^].

### 3.2. Changes in pH and [Hg^2+^] in Aqueous Solution

**Figure 2:**
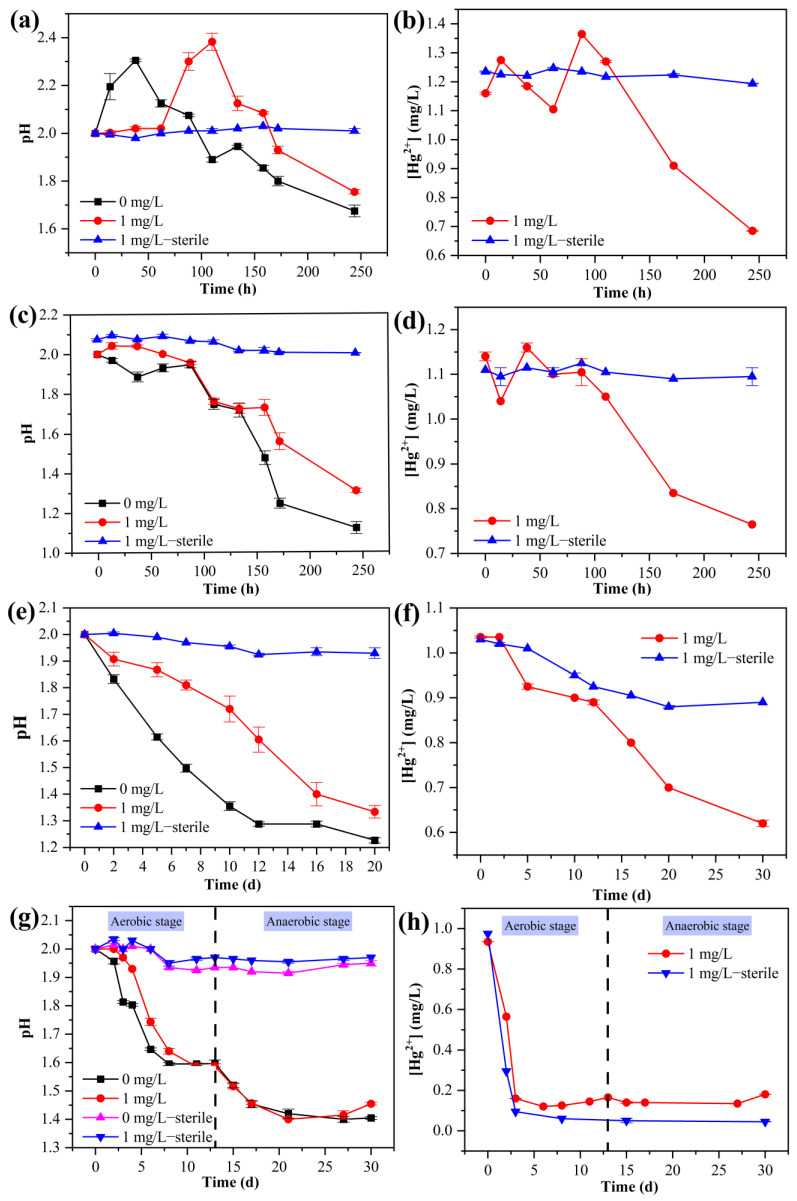
pH value and [Hg^2+^] for the groups of *A. ferrooxidans* ATCC 23270 grown with Fe^2+^ (**a**,**b**), S^0^ (**c**,**d**), anaerobic Fe^3+^ reduction coupled with S^0^ oxidation (**e**,**f**), and aerobic–anaerobic coupling (**g**,**h**) with and without the addition of 1 mg/L Hg^2+^.

For the group with Fe^2+^ oxidation by *A. ferrooxidans* ATCC 23270 under aerobic conditions (Figure 2a and Figure S2d), the overall pH value increased initially and then decreased. Compared with the bio group without Hg^2+^, the pH value of the bacterial experimental group with 1 mg/L [Hg^2+^] increased and decreased slowly, and the lowest pH value (1.76) was relatively high. [Hg^2+^] in the solution for the bio group with Hg^2+^ showed an overall downward trend, and [Hg^2+^] fluctuated with time during 0–88 h and rapidly decreased after 88 h (Figure 2b). This may have been because *A. ferrooxidans* ATCC 23270 can reduce the total Hg concentration by reducing Hg^2+^ to Hg^0^. In addition, the formation of ferrosite substances also has a certain adsorption effect on Hg [24]. In contrast, there was no significant change in [Hg^2+^] for the sterile control group.

For the groups with S^0^ oxidation by *A. ferrooxidans* ATCC 23270 under aerobic conditions (Figure 2c and Figure S2e), the pH value of the solution showed a decreasing trend with time within 0–244 h. There was no obvious change in pH value for the bacterial group within 0–88 h. The overall pH value decreased slowly and finally reached the lowest pH value (244 h). *A. ferrooxidans* uses S^0^ as the energy substrate for oxidation, which is an acid-producing process, resulting in a decrease in solution pH value. However, Hg^2+^ can obviously inhibit the process, resulting in a slower pH decline in the group with Hg^2+^. There was no significant change in the sterile control group [Hg^2+^] (Figure 2d). For the bacterial group with [Hg^2+^], the effect of mercury stress was the weakest when the concentration of Hg^2+^ reached 0.83 mg/L. Hg^2+^ in the solution reacts with SO_4_^2−^ to form HgSO_4_, which can also be complexed with S^0^ and adsorbed to the surface of S^0^ particles [25]. It can also bind Hg^2+^ through sulfhydryl groups on the surface of bacteria, resulting in a decrease in [Hg^2+^] in the solution.

For the group with the anaerobic Fe^3+^ reduction coupled with S^0^ oxidation by *A. ferrooxidans* ATCC 23270 (Figure 2e and Figure S2f), the overall pH value showed a downward trend. The pH value of the group without Hg^2+^ decreased rapidly and reached the lowest value (1.28) at day 12. Compared with the bacterial group with 1 mg/L [Hg^2+^], the pH value decreased more slowly and reached the lowest level at day 20 (1.33). There was no significant change in pH value over time for the sterile group with Hg^2+^. As seen from the pH change trend for each system, anaerobic Fe^3+^ coupled with S^0^ oxidation is an acid-producing process, and the presence of Hg^2+^ has a certain inhibitory effect on its growth and metabolism. The bacterial group with Hg^2+^ showed a decreasing trend (Figure 2f). When [Hg^2+^] was 0.8 mg/L, the stress effect on *A. ferrooxidans* ATCC 23270 was the weakest. In contrast, there was no significant change in [Hg^2+^] for the sterile group.

For the aerobic–anaerobic coupling group (Figure 2g and Figure S2g), the overall pH value decreased and showed a gentle trend over time during the aerobic stage (0–13 d) due to bacterial iron and sulfur oxidation. After entering the anaerobic stage (13–30 d), the pH value of the solution further dropped to the lowest value and then reached stability. At the same time, the pH value of the sterile system decreased slightly due to abiotic oxidation, but there was no significant change trend. In the aerobic culture stage, the pH value of the bacterial group with 1 mg/L [Hg^2+^] decreased more slowly than that of the bacterial group without Hg^2+^ and reached the lowest pH value (1.58) at day 12. There was no significant difference in pH value in the anaerobic culture stage. In addition, the overall pH of the sterile control group with Hg^2+^ was higher than that without Hg^2+^. For the group with Hg^2+^, [Hg^2+^] showed a rapid decline and then a stable trend (Figure 2h). Compared with the sterile group, [Hg^2+^] decreased slower and was higher for the bio group, indicating that pyrite enables the strong adsorption of Hg^2+^. Due to the Hg transformation of bacteria, Hg exists in the solution for a long time [26]. With the utilization of pyrite by *A. ferrooxidans* ATCC 23270, a small amount of Hg^2+^ returns to the solution through the mercury transformation of bacteria [27], resulting in the fluctuation of [Hg^2+^] in the solution for the bio groups over time. When [Hg^2+^] is 1 mg/L, the stress effect produced by *A. ferrooxidans* ATCC 23270 is the strongest. In the process of bacterial growth, [Hg^2+^] floats within the range of 0.15 ± 0.03 mg/L and finally reaches [Hg^2+^]_min_ of 0.14 mg/L in the anaerobic stage (Figure 1g).

### 3.3. Changes in Cell Morphology and Element Distribution

**Figure 3:**
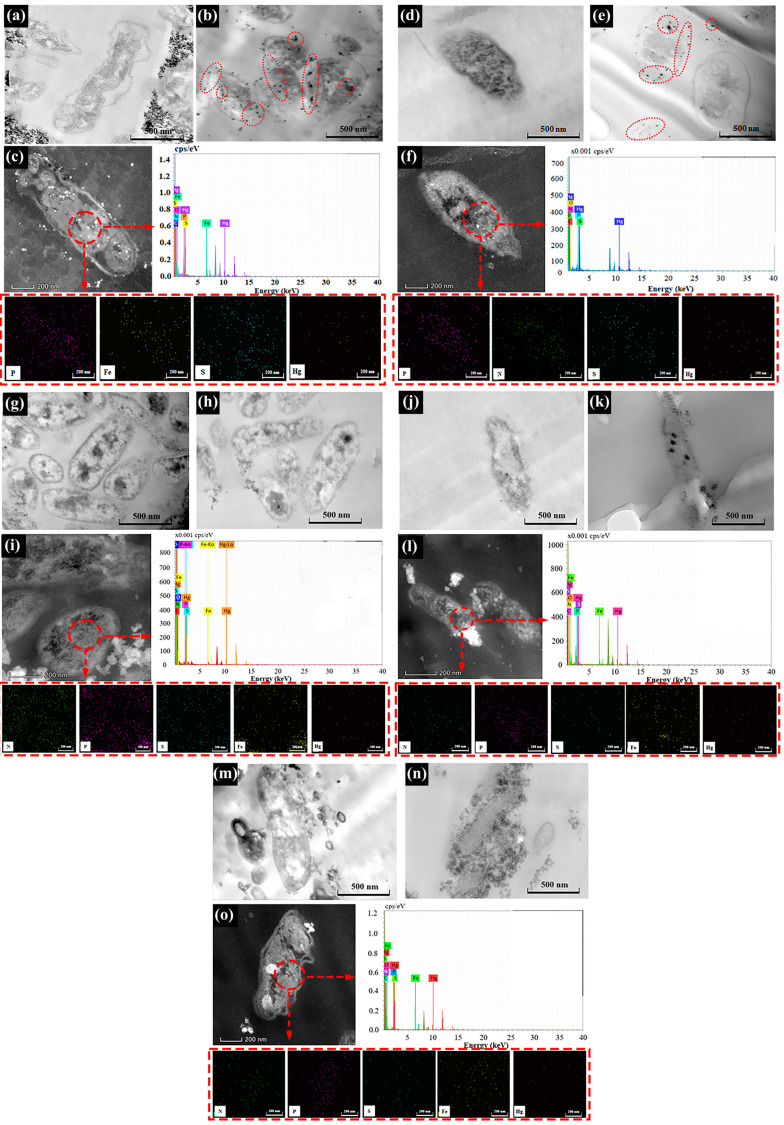
TEM images and TEM-EDS images of bacterial cells of *A. ferrooxidans* ATCC 23270 in the presence of Hg^2+^. (**a**) TEM images for the group with Fe^2+^ oxidation with 0 mg/L [Hg^2+^]; (**b**) TEM images for the group with Fe^2+^ oxidation with 1 mg/L [Hg^2+^]; (**c**) TEM-EDS images for the group with Fe^2+^ oxidation with 1 mg/L [Hg^2+^]; (**d**) TEM images for the group with S^0^ oxidation with 0 mg/L [Hg^2+^]; (**e**) TEM images for the group with S^0^ oxidation with 1 mg/L [Hg^2+^]; (**f**) TEM-EDS images for the group with S^0^ oxidation with 1 mg/L [Hg^2+^]; (**g**) TEM images for the group with anaerobic Fe^3+^ reduction coupled with S^0^ oxidation with 0 mg/L [Hg^2+^]; (**h**) TEM images for the group with anaerobic Fe^3+^ reduction coupled with S^0^ oxidation with 1 mg/L [Hg^2+^]; (**i**) TEM-EDS images for the group with anaerobic Fe^3+^ reduction coupled with S^0^ oxidation with 1 mg/L [Hg^2+^]; (**j**) TEM images for the group with the aerobic stage of aerobic–anaerobic coupling with 0 mg/L [Hg^2+^]; (**k**) TEM images for the group with the aerobic stage of aerobic–anaerobic coupling with 1 mg/L [Hg^2+^]; (**l**) TEM-EDS images for the group with the aerobic stage of aerobic–anaerobic coupling with 1 mg/L [Hg^2+^]; (**m**) TEM images for the group with the anaerobic stage of aerobic–anaerobic coupling with 0 mg/L [Hg^2+^]; (**n**) TEM images for the group with the anaerobic stage of aerobic–anaerobic coupling with 1 mg/L [Hg^2+^]; (**o**) TEM-EDS images for the group with the anaerobic stage of aerobic–anaerobic coupling with 1 mg/L [Hg^2+^].

*A. ferrooxidans* ATCC 23270 cells of different sections for the Fe^2+^ oxidation group showed clear cell boundary outlines and cell content shadows (Figure 3a,b). Black spots appeared in the cell sections for the group with Fe^2+^ oxidation with Hg^2+^, and the black spots were distributed outside the cells. The main components inside the cells were N, O, S, and Fe. The bacterial group with Hg^2+^ had a small amount of Hg inside the cells (Figure 3c). In addition, the proportion of Fe in the group with Hg^2+^ was higher than in that without Hg^2+^, but the proportion of S was lower than in that without Hg^2+^ (Figure S3a). Through the analysis of the distribution of elements in the cells of the Fe^2+^ oxidation group, it was found that the main element composition of the black particle region in the cells of the group with Hg^2+^ was probably Fe. Since no additional S^0^ was added in the Fe^2+^ oxidation group, the bacteria had a weak ability to enrich Hg^2+^, resulting in low Hg content in bacterial cells, which were difficult to detect.

For the group with S^0^ oxidation with Hg^2+^, the cross-section of cells shows numerous small black spots on the outside and inside of the cell wall (Figure 3d,e). In addition to N, O, S, P, and other elements, Hg was also detected at a higher proportion in the cells for the bio group with Hg^2+^ (Figure 3f). In addition, the proportion of S was significantly higher here than in the group without Hg^2+^ (Figure S3b). Comprehensive analysis of the composition of elements inside cells in the S^0^ oxidation group showed that a large amount of Hg elements were distributed in the black particle area inside the cells of the group with Hg^2+^, which further proved that Hg^2+^ was captured by bacteria through the extracellular polymer and transported to the cell for transformation.

For the anaerobic Fe^3+^ reduction group, the cell outline was clear, and the contents were obviously shaded (Figure 3g,h). Here, there were a few black particles in the middle of the cell walls of the cells without Hg^2+^ (Figure 3g). Under the influence of Hg^2+^, a small amount of black granular material was adsorbed on the cell surface, and a shadow of flocculent material was evident around the cell (Figure 3h). The cell interior was mainly composed of N, O, S, P, Fe, and other elements (Figure 3i). The proportion of N and O elements in cells supplemented with Hg^2+^ was significantly higher than that in cells without Hg^2+^, indicating that cells would secrete more organic substances under the influence of Hg^2+^ (Figure S3c). In addition, a very small amount of Hg was detected inside the cells of the system supplemented with Hg^2+^, and it is possible that most Hg was mainly distributed on the cell surface. Through comprehensive analysis of the above results, it was found that the black particles in the cells without Hg^2+^ addition may have been Fe, while the black particles on the cell wall surface in the system with Hg^2+^ addition may have been related to Hg, which further proves that *A. ferrooxidans* ATCC 23270 can capture Hg^2+^ through the extracellular polymer on the cell surface. In addition, under the influence of Hg^2+^, *A. ferrooxidans* ATCC 23270 may secrete more carbohydrates, lipids, proteins, and other substances for the mercury conversion process.

For the aerobic–anaerobic coupling group, in the aerobic phase, there were large black particles inside the bacterial cells and outside the cell wall with the addition of Hg^2+^ (Figure 3j,k). The main components of the cells were N, P, O, S, and Fe (Figure 3l). The proportion of N and P elements in bacteria without Hg^2+^ was significantly higher than that in bacteria with Hg^2+^ under aerobic conditions (Figure S3d). In addition, the bacterial group with Hg^2+^ had a higher proportion of Fe. Combined with TEM analysis, it was found that the black particles in the cells were likely to be Fe complexes. This result was consistent with the change in [Fe^3+^] in the bacterial leaching process (Figure 1h), indicating that the metabolic activity of bacteria was significantly improved after the removal of Hg stress under the addition of Hg^2+^. There was more flocculent material around the cells growing in the anaerobic stage (Figure 3m,n). After entering the anaerobic stage, the proportion of N and O elements in bacteria in the bacterial group with Hg^2+^ increased significantly, which was almost the same as the result for the bacteria without Hg^2+^ (Figure 3o and Figure S3e). This result indicates that the Hg^2+^ stress effect on bacteria was greatly reduced at this stage. In the bacterial system with added Hg^2+^, only a very small amount of Hg was detected inside the cells under aerobic and anaerobic conditions (Figure 3o), possibly because most of the added Hg^2+^ was adsorbed on the surface of pyrite, so the concentration of Hg on the surface and inside the cells was very low.

### 3.4. Changes in Solid Residue Morphology and Element Distribution

**Figure 4:**
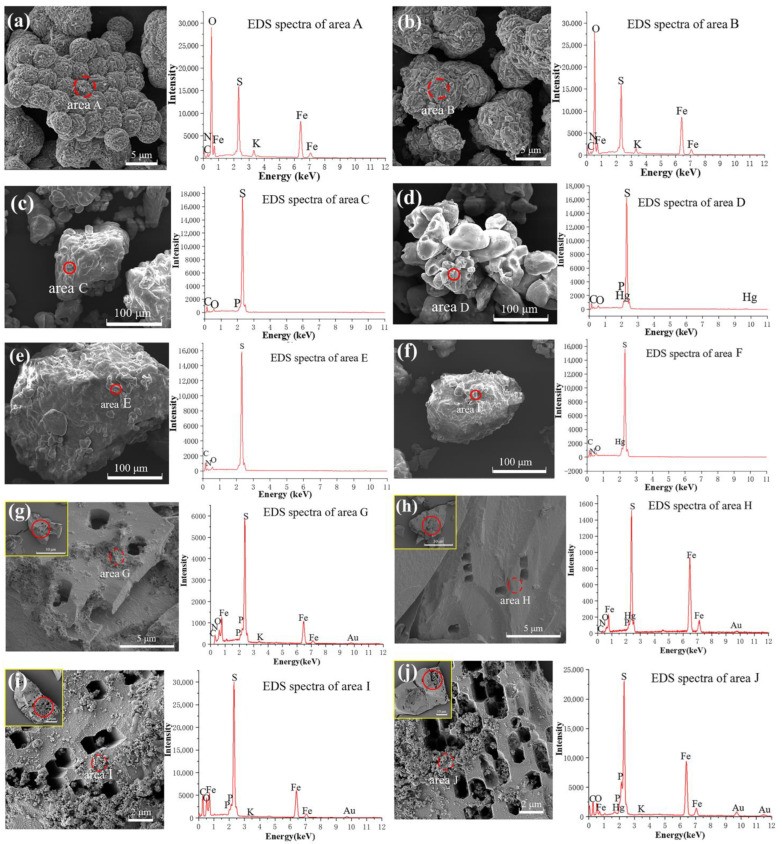
SEM-EDS images of bacterial cells for *A. ferrooxidans* ATCC 23270 in the presence of Hg^2+^. (**a**) Fe^2+^ oxidation with 0 mg/L [Hg^2+^]; (**b**) Fe^2+^ oxidation with 1 mg/L [Hg^2+^]; (**c**) S^0^ oxidation with 0 mg/L [Hg^2+^]; (**d**) S^0^ oxidation with 1 mg/L [Hg^2+^]; (**e**) anaerobic Fe^3+^ reduction coupled with S^0^ oxidation with 0 mg/L [Hg^2+^]; (**f**) anaerobic Fe^3+^ reduction coupled with S^0^ oxidation system with 1 mg/L [Hg^2+^]; (**g**) anaerobic Fe^3+^ reduction coupled with S^0^ oxidation with 1 mg/L [Hg^2+^]; (**h**) the aerobic stage of aerobic– anaerobic coupling with 0 mg/L [Hg^2+^]; (**i**) the aerobic stage of aerobic–anaerobic coupling with 1 mg/L [Hg^2+^]; (**j**) the anaerobic stage of aerobic–anaerobic coupling with 0 mg/L [Hg^2+^].

The SEM-EDS results for the substrate residue at 244 h in the *A. ferrooxidans* ATCC 23270 Fe^2+^ oxidation process are shown in Figure 4a,b. There were no significant differences in the morphologies of the substrate residues in the two systems, and the residues were agglomerated into spheroids with sharp edges and good crystals (Figure 4a,b). The main components of the substrate residue were O, Fe, S, and K. The results show that there was no significant change in the proportion of the main components of the substrate residue in the Hg^2+^ system compared with that in the system with addition, and no Hg was detected. In summary, the above results indicate that the main component of the substrate residue formed in the Fe^2+^ oxidation group may have been jarosite. In addition, the reason why Hg could not be detected in the substrate residue of the group with Hg^2+^ may have been that the EDS could not be detect it due to the low content of Hg adsorbed on the surface of jarosite. Further detection approaches are needed to analyze the Hg element on the jarosite surface.

In the sulfur oxidation group, the substrate residue surface in the system without Hg^2+^ was obviously eroded by bacteria and the surface was rough, but the elemental sulfur particles were intact (Figure 4c). In addition, compared with the bacterial group, the substrate residues of the Hg^2+^ system showed smaller particle sizes and less aggregation, with fewer surface pits but a greater depth (Figure 4d). The EDS results show that the substrate residue surface was still dominated by S, and trace Hg was detected on the surface of the Hg^2+^ system. In conclusion, bacteria in the group without Hg^2+^ were mainly attached to the surface of sulfur for erosion, thus leaving dense indentations, while the group with Hg^2+^ may have eroded into the interior of the sulfur, resulting in fewer but deeper indentations on the surface of the substrate residue. In addition, Hg^2+^ in solution can be transformed and adsorbed to the surface of sulfur under the action of bacteria and can also combine with the surface of sulfur through chemical action.

For the anaerobic Fe^3+^ reduction coupled with S^0^ oxidation group, the substrate residue of the system without Hg^2+^ was rough, uneven, and covered with pits, and more granular debris was attached (Figure 4e). This may have been caused by bacterial erosion. There were fine cracks and more pits on the surface of the substrate residue in the group with Hg^2+^ added, as well as smaller fragments attached to the surface, and the whole surface was looser (Figure 4f). Compared with the substrate residue without Hg^2+^, the degree of bacterial erosion was slightly less, indicating that the presence of Hg^2+^ had an effect on the growth and metabolism of bacteria. Since only S^0^ was added to the solid substrate, EDS analysis showed that the main components of the substrate residue were S followed by C, O, and a small amount of Fe. A large proportion of Hg was detected on the substrate residue surface of the Hg^2+^ system (Figure 4f), indicating that the Hg transformation mechanism of *A. ferrooxidans* ATCC 23270 helps to transfer Hg^2+^ in solution to the substrate residue surface and fix it by combining it with S.

In the aerobic and anaerobic coupled system, pits caused by bacterial erosion appeared on the mineral surface at the aerobic stage, and a small amount of flocculent substance was present around the pits (Figure 4g,h). After a period of anaerobic culture, patches of regularly shaped erosion pits appeared on the mineral surface, accompanied by a large amount of flocculent material (Figure 4i,j). The EDS results show that the proportions of C, N, O, P, and other elements in substrate residues were slightly higher, indicating that organic substances such as proteins, lipids, and sugars may be secreted on the surface of minerals during the interaction between bacteria and minerals. The proportion of C element (22.96%, 45.64%) on the substrate residue surface of the system without Hg^2+^ addition in the aerobic stage and anaerobic stage was significantly higher than that in the system with Hg^2+^ addition (0.64%, 32.07%), indicating that the flocculent material around the bacterial erosion pit was organic material composed of C. The addition of Hg^2+^ had a strong inhibitory effect on the growth and metabolism of bacteria. In addition, the proportion of Hg on the surface decreased gradually from the aerobic stage to the anaerobic stage until no Hg was detected at the end of the anaerobic stage, indicating that the interaction between bacteria and minerals accompanied the mercury transformation process.

### 3.5. Changes in Morphology, Composition, and Phase of Solid Residue

**Figure 5:**
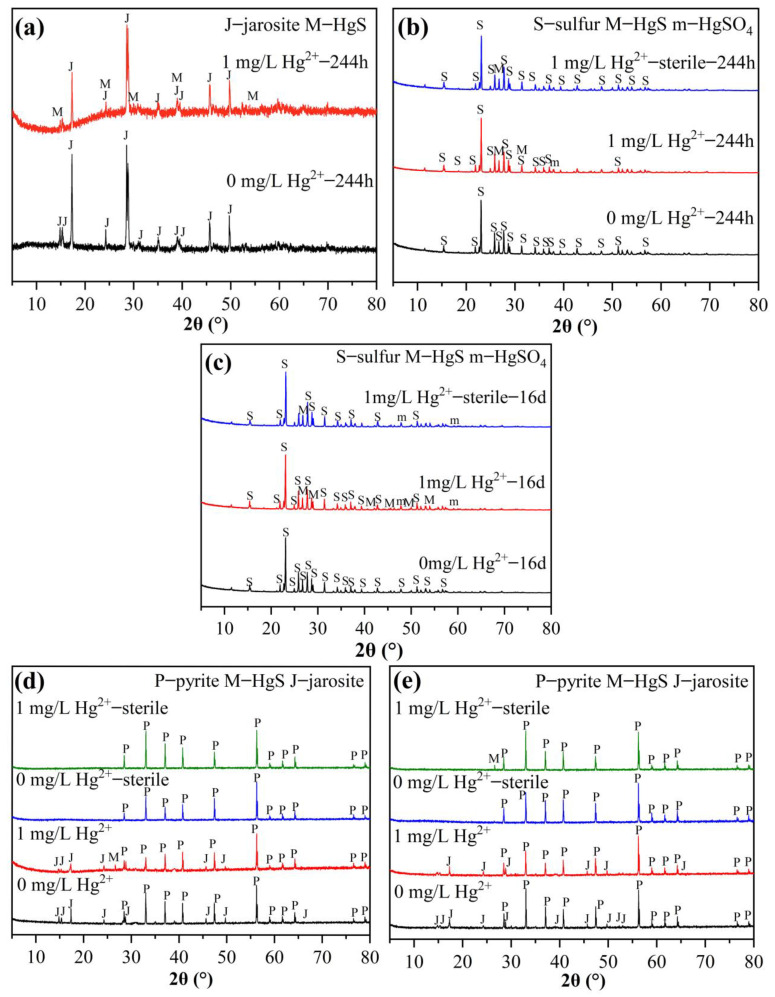
XRD images of bacterial cells of *A. ferrooxidans* ATCC 23270 in the presence of Hg^2+^. (**a**) Fe^2+^ oxidation; (**b**) S^0^ oxidation; (**c**) anaerobic Fe^3+^ reduction coupled with S^0^ oxidation; (**d**) the aerobic stage of aerobic–anaerobic coupling; (**e**) the anaerobic stage of aerobic–anaerobic coupling.

XRD was used to characterize substrate residues in different culture systems, and the results are shown in Figure 5. The XRD results for the substrate residue for the group with Fe^2+^ oxidation (Figure 5a) show that the main component of the solid substance was jarosite when cultured to 244 h, indicating that the Fe^2+^ in the bacterial oxidation solution produced a large amount of Fe^3+^, which reacted with SO_4_^2−^ and K^+^ in the solution to produce jarosite precipitation [28]. A small amount of HgS was found for the group with the addition of Hg^2+^. The results for the group with S^0^ oxidation are shown in Figure 5b. The main component of the substrate residue was still mainly S^0^, indicating that, because the products of bacterial oxidation of S^0^ are mainly water-soluble, there was little residue on the surface, and it could not be detected. In addition, the XRD results for the substrate residues in each system showed no significant differences in peak shape and intensity, and it was found that diffraction peaks related to HgS and HgSO_4_ basically coincided with S^0^ diffraction peaks, so it was impossible to accurately judge whether Hg was present on the surfaces of substrate residues in the Hg^2+^ system. For the group with anaerobic Fe^3+^ reduction coupled with S^0^ oxidation, the main component of the substrate residue in different systems was still S^0^ at day 16 (Figure 5c). Due to the low concentration of Hg^2+^ applied and the fact that most of the XRD peaks of mercury compounds coincide with the characteristic peaks of S^0^, the phase changes on the substrate residue surface could not be accurately characterized (Figure 5c).

For the aerobic–anaerobic coupling group, the substrate residue was mainly composed of pyrite at day 13 of cultivation under aerobic conditions, and a small jarosite diffraction peak appeared in the bio group (Figure 5d). After entering the anaerobic environment for a period of time, the main components of the substrate residue for the bio groups were still pyrite and a small amount of jarosite, but the intensity of the jarosite diffraction peak decreased significantly, indicating that the anaerobic conditions under the action of bacteria may lead to partial jarosite dissolution (Figure 5e). In addition, the HgS diffraction peak appeared in the same position in the bacterial system with Hg^2+^ at day 13 and the sterile system with Hg^2+^ at day 30, which was consistent with the SEM-EDS results, confirming that the contents of Hg on the substrate residue surfaces of the bacterial system and the sterile system were different. This indicates that *A. ferrooxidans* ATCC 23270 mercury transformation can lead to the migration of mercury elements between the surface of the substrate residue and the solution.

**Figure 6:**
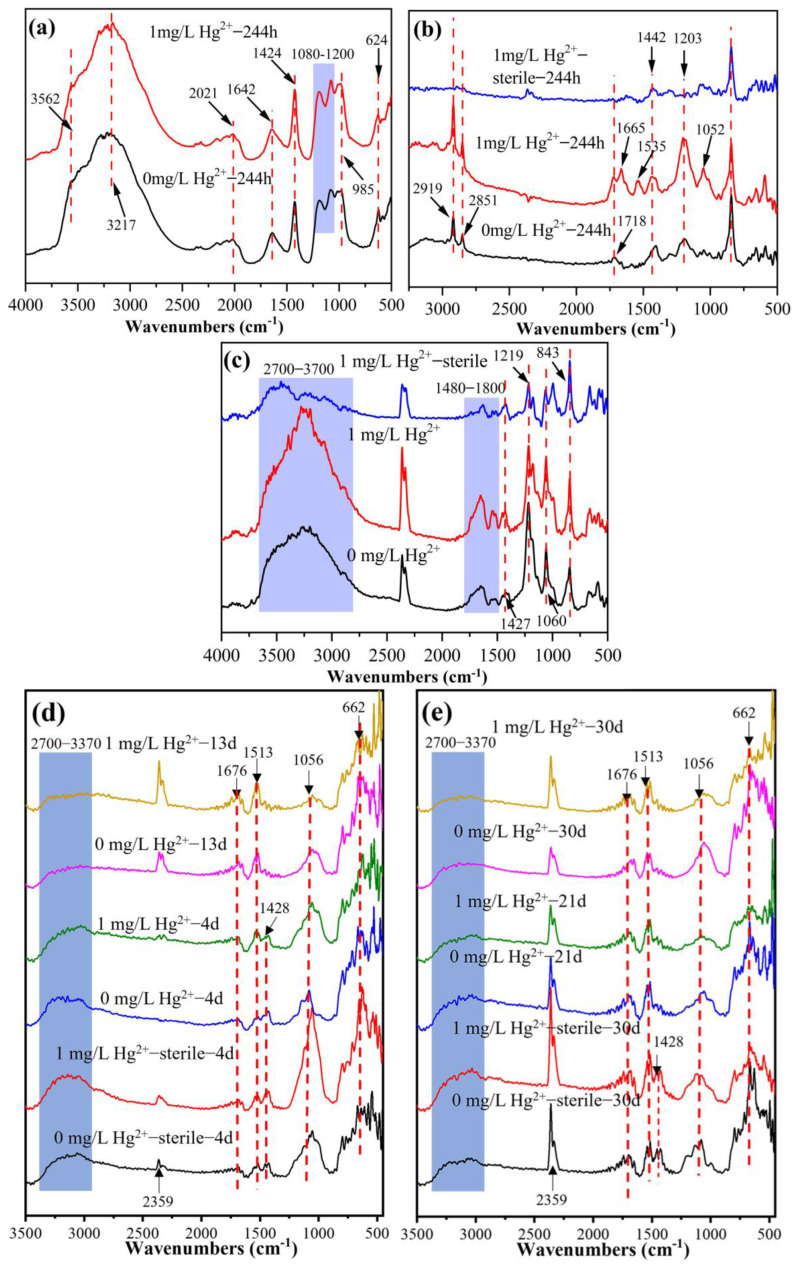
FTIR images of bacterial cells of *A. ferrooxidans* ATCC 23270 in the presence of Hg^2+^. (**a**) Fe^2+^ oxidation; (**b**) S^0^ oxidation; (**c**) anaerobic Fe^3+^ reduction coupled with S^0^ oxidation; (**d**) the aerobic stage of aerobic–anaerobic coupling; (**e**) the anaerobic stage of aerobic–anaerobic coupling.

To explore the compositions of and changes in substrate residues on the surface of *A. ferrooxidans* ATCC 23270 cells, FT-IR spectroscopy was undertaken according to a methodology from the literature [29,30], and the results are shown in Figure 6. In the group with Fe^2+^ oxidation by *A. ferrooxidans* ATCC 23270, sharp absorption peaks were found at 624, 985, 1080–1200, 1424, and 1642 cm^−1^, and wide and strong absorption peaks were found at 2021, 3217, and 3561 cm^−1^ (Figure 6a). Among these, the peaks at 624 and 1080–1200 cm^−1^ represent the absorption of PO_2_^−^ on the surface of phosphate groups or phospholipids [31] or were generated by SO_4_^2−^ stretching vibration [32]. The absorption peak at 985 cm^−1^ represents the deformation vibration of OH, and the absorption peak at 1642 cm^−1^ represents the deformation vibration of HOH [33,34]. The range from 3200 to 3600 cm^−1^ is the vibration absorption of -OH, and the range from 1424 cm^−1^ is the absorption of -CH_3_, indicating the presence of lipid or carbohydrate adsorption. The *A. ferrooxidans* ATCC 23270 Fe^2+^ oxidation system formed in the substrate residue was the main component of jarosite.

In the S^0^ oxidation system, the substrate residue surface of the bacterial group with Hg^2+^ showed higher absorption peaks at 1052, 1203, 1442, 1535, 1665, and 1720 cm^−1^ than in that without Hg^2+^ (Figure 6b). The peak at 1052 cm^−1^ represents the vibration absorption of S=O, the peaks at 1203 and 1442 cm^−1^ represent the absorption generated by the bending vibration of -CH_2_ and -CH_3_ in lipids, and the peaks at 1535 and 1650–1850 cm^−1^ represent the absorption peak generated by the vibration of -NH_2_ and -C=O. These results indicate that the substrate residues in the bacterial group with Hg^2+^ formed a large number of organic components, such as proteins and lipids, after bacterial action at 244 h. It was found that the bacteria in the group without Hg^2+^ began to adsorb and grow on the substrate sulfur at the early stage (14 h), reached the highest metabolic intensity at the middle stage (110 h), and entered the declining stage later (244 h), and the amount of organic matter adsorbed on the surface of the substrate residue decreased. However, the bacteria in the group with Hg^2+^ grew slowly in the early and middle stages (14 h, 110 h) due to the inhibiting effect of Hg^2+^. After the bacteria gradually came to tolerate Hg^2+^ through the mechanism of mercury conversion, Hg^2+^ stress was finally relieved, and the growth and metabolism of bacteria reached the most vigorous stage (Figure 6b and Figure S4a,b).

A large number of absorption peaks with different strengths were present on the substrate residue surface at 500–2000 cm^−1^, among which 843 cm^−1^ is the characteristic peak of S^0^ [35], 1060 cm^−1^ is the absorption peak generated by S=O vibration, and 1219 cm^−1^ is the absorption peak of the C-O bond (Figure 6c). The absorption peak of carbohydrate or lipid -CH_3_ groups is 1427 cm^−1^; 1480–1800 cm^−1^ is the absorption peak generated by the vibration of protein amide bonds; 2700–3700 cm^−1^ is a strong and wide peak and is considered to be related to -OH and -NH. It was found that the peak intensity of the bacterial system in the carbohydrate-, lipid-, and protein-related absorption peak region was significantly higher than that of the sterile group, indicating that the bacterial growth and metabolism intensity in the system was high, and there was a greater amount of adsorbed bacteria on the substrate residue surface.

All the substrate residues showed different intensity absorption peaks at 662, 1056, 1428, 1513, 1676, 2359, and 2700–3370 cm^−1^ (Figure 6d,e). Here, 662 and 1056 cm^−1^ represent the absorption peaks generated by the bending vibration and asymmetric stretching vibration of SO_4_^2−^, 1428 cm^−1^ represents the absorption peak of -CH_3_, and 1513 and 1676 cm^−1^ represent the absorption peaks related to amide bonds in proteins; 2359 cm^−1^ is generally considered to be the absorption peak produced by CO_2_, and the strong and wide peak at 2700–3370 cm^−1^ represents the stretching vibration of the -OH group. It was found that the SO_4_^2−^ (662 and 1056 cm^−1^), -CH_3_ (1428 cm^−1^), and -OH (2700–3370 cm^−1^) absorption peaks on the substrate residue surface of the sterile control group were stronger, and the SO_4_^2−^ absorption peaks decreased with the increase in time. After bacterial action, the -CH_3_- and -OH-related absorption peaks gradually decreased with the increase in time, while the protein-related absorption peaks at 1513 and 1676 cm^−1^ were significantly enhanced, indicating that bacterial action resulted in changes in the crystal structure of the mineral surface, increased the adsorption of organic matter on the substrate residue surface, and made the bacterial metabolism more vigorous.

**Figure 7:**
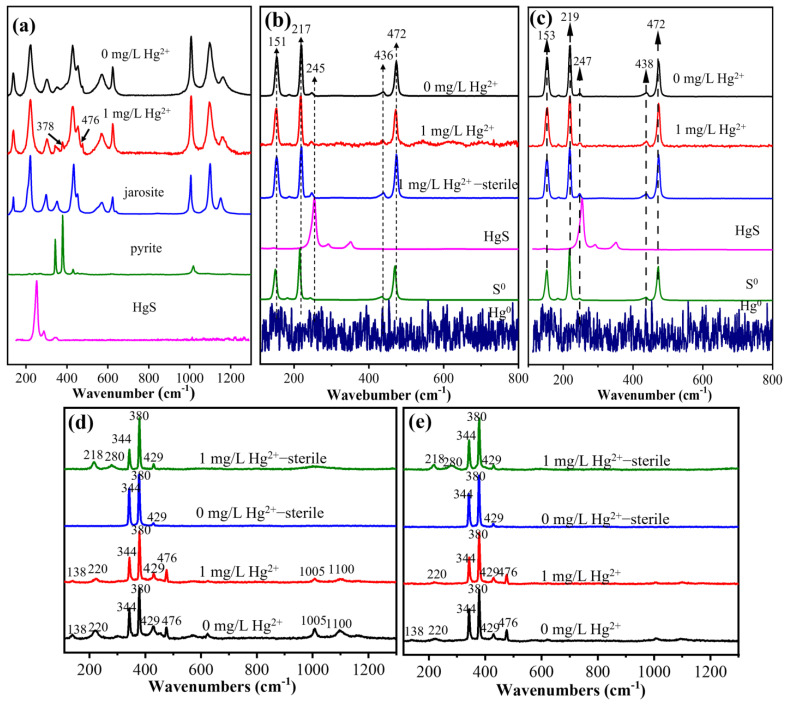
Raman images of bacterial cells of *A. ferrooxidans* ATCC 23270 in the presence of Hg^2+^. (**a**) Fe^2+^ oxidation; (**b**) S^0^ oxidation; (**c**) anaerobic Fe^3+^ reduction coupled with S^0^ oxidation; (**d**) the aerobic stage of aerobic–anaerobic coupling; (**e**) the anaerobic stage of aerobic–anaerobic coupling.

The Raman spectra show that the main component of the substrate residue for the group with Fe^2+^ oxidation by *A. ferrooxidans* ATCC 23270 was jarosite (Figure 7a). The substrate residue of the bacterial system with Hg^2+^ had an obvious and sharp absorption peak at 378 cm^−1^, which was FeS_2_, indicating that the addition of Hg^2+^ leads to the formation of Fe-S bonds on the surface of the jarosite. The substrate residues for the groups with aerobic S^0^ oxidation and anaerobic Fe^3+^ reduction coupled with S^0^ oxidation had obvious S^0^ peaks at 151, 217, 245, 436, and 472 cm^−1^ (Figure 7b,c). For the bio group with Hg^2+^, the substrate residue showed obvious signal noise, indicating the presence of Hg^0^ on the surface of the substrate residue. This then continued to transform into other forms of Hg.

In the Raman spectra for the aerobic–anaerobic coupling group, the pyrite characteristic peaks were found at 344, 380, and 429 cm^−1^ (Figure 7d,e). In addition, the characteristic peaks of jarosite appeared at 138, 220, 1005, and 1100 cm^−1^, and the characteristic peaks of jarosite (Figure 7d) in the aerobic stage were stronger than those in the anaerobic stage (Figure 7e). At the same time, the characteristic peaks of S_n_^2−^ and other polysulfides produced during the oxidation of pyrite appeared at 476 cm^−1^, which indicates that the surface of the mineral was oxidized by bacteria and formed jarosite and polysulfides [36]. In addition, the sterile control group with Hg^2+^ showed the characteristic peaks of Fe-O in jarosite and Hg-S in HgS at 218 and 280 cm^−1^, indicating that Hg^2+^ in the solution mainly combined with S on the surface of pyrite to form Hg-S bonds and affected the structure of surface minerals.

**Figure 8:**
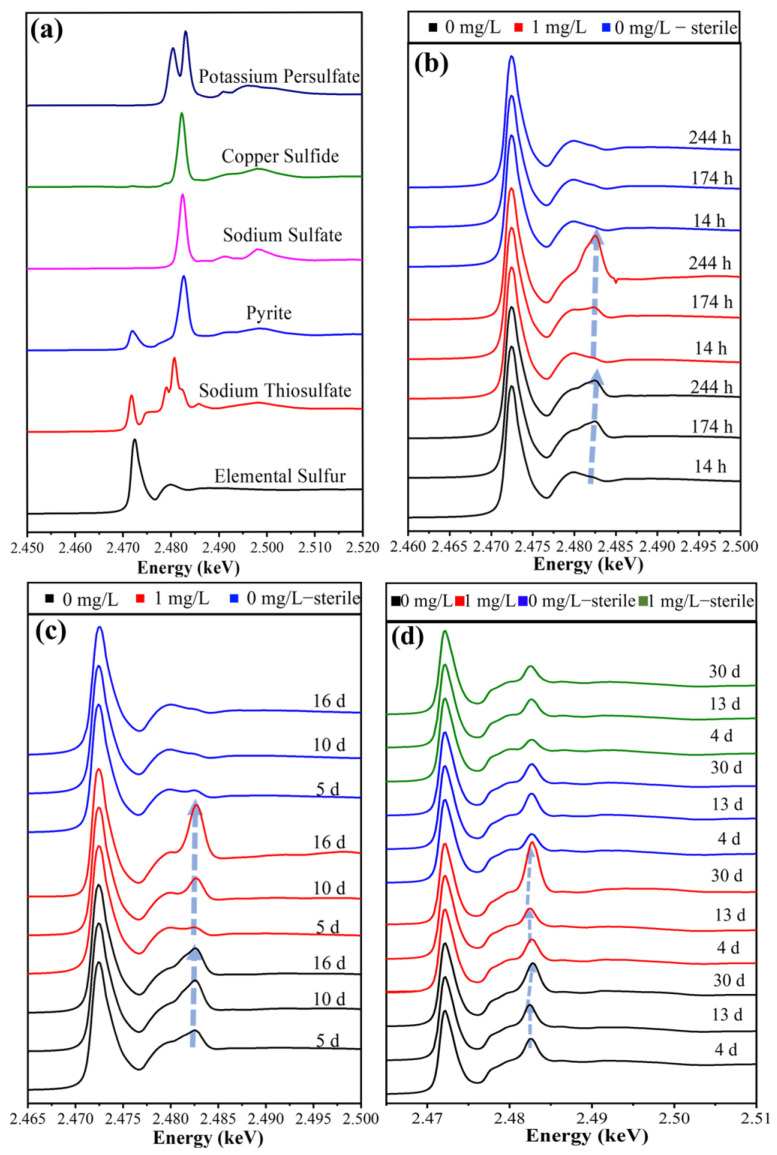
XANES images of bacterial cells of *A. ferrooxidans* ATCC 23270 in the presence of Hg^2+^. (**a**) reference samples; (**b**) S^0^ oxidation; (**c**) anaerobic Fe^3+^ reduction coupled with S^0^ oxidation; (**d**) aerobic–anaerobic coupling.

In the S K-edge XANES analysis, elemental sulfur (S^0^), sodium thiosulfate (Na_2_S_2_O_3_), pyrite (FeS_2_), sodium sulfate (Na_2_SO_4_), copper sulfide (CuS), and potassium persulfate (K_2_S_2_O_8_) were selected as S-containing reference samples (Figure 8a). In the S^0^ oxidation group, the characteristic peak of the substrate residue surface S^0^ was at 2.4724 keV, and the characteristic peak of SO_4_^2−^ was at 2.4825 keV (Figure 8b). With the oxidation of S^0^ in the bio group, the surface of substrate S^0^ was oxidized to SO_4_^2−^. In addition, the SO_4_^2−^ characteristic peak signal on the substrate residue surface was the strongest in the bacterial group at 244 h, which was consistent with the FT-IR result relating to the substrate residue (Figure 6b), confirming that Hg^2+^ may be present in the substrate residue in the form of HgSO_4_.

With the interaction between *A. ferrooxidans* ATCC 23270 and substrate S^0^, the substrate residue surface showed a strong absorption peak at 2.4825 keV, which gradually increased with time (Figure 8c). It was found that the sulfur speciation transformed from S^0^ to SO_3_^2−^ and SO_4_^2−^, indicating that the substrate S^0^ in each group was gradually oxidized. Compared with the bacterial group without Hg^2+^, the SO_4_^2−^ absorption peak intensity was lower at day 5 and day 10, while the peak intensity was significantly increased at day 16. The results indicate that the bacteria were strongly inhibited by Hg^2+^ in the early and middle stages of growth, and the growth and metabolic intensity were significantly improved after the inhibition of Hg^2+^ was lifted in the later stage.

The characteristic peaks of FeS_2_ and SO_4_^2−^ were located at 2.4721 eV and 2.4826 eV, respectively (Figure 8d). With time, in the bacterial system, the absorption peak at SO_4_^2−^ shifted to the right to 2.4828 eV, and the peak intensity continued to increase, indicating that the S species on the substrate residue surface were continuously oxidized, with the formation of jarosite and other intermediates. In addition, the SO_4_^2−^ absorption peak intensity on the substrate residue surface for the bio group with Hg^2+^ was much higher than that of other systems at day 30, which was the same result as in Figure 8b,c, indicating that the metabolic activity of bacteria was greatly improved after the removal of the inhibition of Hg^2+^.

Via the comprehensive analysis of the XPS spectral fitting results for the substrate residue surface Hg 4*f* (see Figure S4 and the relevant description in the Supplementary Materials) and the S K-edge XANES spectra for all experimental groups, it was found that the morphological transformations of S, Fe, and Hg are closely related to the Fe/S oxidation of *A. ferrooxidans* ATCC 23270. *A. ferrooxidans* ATCC 23270 oxidizes the substrate, then the S/Fe morphology changes, and SO_4_^2−^/Fe^3+^ is generated.

### 3.6. Transcriptome Analysis

**Figure 9:**
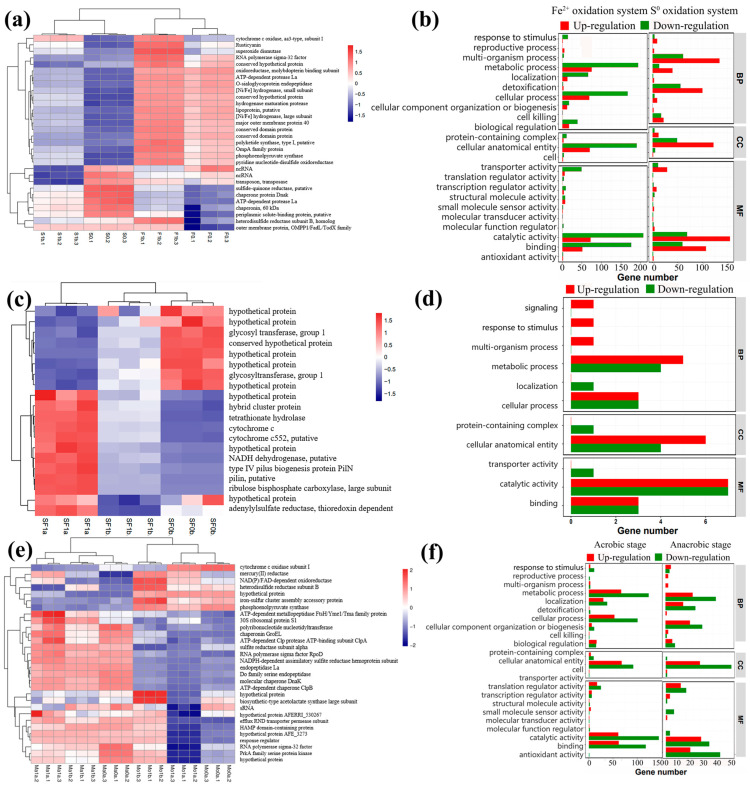
Heatmap of expression and GO analysis of DEGs by *A. ferrooxidans* ATCC 23270 in the presence of Hg^2+^. (**a**) Heatmap of expression during the oxidation of S^0^/Fe^2+^ by *A. ferrooxidans* ATCC 23270 in the presence of Hg^2+^. (**b**) GO analysis of DEGs during the oxidation of S^0^/Fe^2+^ by *A. ferrooxidans* ATCC 23270 in the presence of Hg^2+^ (left: Fe^2+^ oxidation system 1 mg/L [Hg^2+^] vs. 0 mg/L [Hg^2+^]; right: S^0^ oxidation system 1 mg/L [Hg^2+^] vs. 0 mg/L [Hg^2+^]). (**c**) Heatmap of expression during the anaerobic Fe^3+^ reduction coupled with S^0^ oxidation of *A. ferrooxidans* ATCC 23270 in the presence of Hg^2+^. (**d**) GO analysis of DEGs during the anaerobic Fe^3+^ reduction coupled with S^0^ oxidation of *A. ferrooxidans* ATCC 23270 in the presence of Hg^2+^ (1 mg/L [Hg^2+^] at the last stage vs. 0 mg/L [Hg^2+^]). (**e**) Heatmap of expression during the *A. ferrooxidans* ATCC 23270–pyrite interaction in the presence of Hg^2+^. (**f**) GO analysis of DEGs during *A. ferrooxidans* ATCC 23270–pyrite interaction in the presence of Hg^2+^ (left: 1 mg/L [Hg^2+^] at the last stage of the aerobic phase vs. 0 mg/L [Hg^2+^] at the aerobic phase; right: 1 mg/L [Hg^2+^] at the last stage of anaerobic phase vs. 0 mg/L [Hg^2+^] at the anaerobic phase).

In the group with Fe^2+^ oxidation (Figure 9a, Table S1), the expressions of cytochrome *c* oxidase and other functional genes of bacteria in the presence of Hg^2+^ were increased, indicating that the iron oxidation function and antioxidant function were enhanced. The expressions of functional genes, such as outer membrane protein, periplasmic solute binding protein, ATP-dependent protease La, and chaperone protein, were increased. The results indicate that the presence of Hg^2+^ improves the protein transcription and translation efficiency of *A. ferrooxidans* ATCC 23270. In the presence of Hg^2+^, *A. ferrooxidans* ATCC 23270 showed enhanced iron and sulfur oxidation metabolism in the system simply using Fe^2+^/S^0^ as a substrate. For the S^0^ oxidation group, the expression levels of some functional genes were significantly different (Figure 9a, Table S1). The expression levels of functional genes such as peripheral solute binding protein and ncRNA decreased, indicating that *A. ferrooxidans* ATCC 23270 stress in the presence of Hg^2+^ leads to a reduction in protein transcription and translation efficiency. The expressions of cytochrome *c* oxidase, pyridine nucleotide disulfide reductase, and other functional genes increased, while the expression of isodisulfide reductase was not significantly different. Thioneone reductase is involved in the reaction between hydrogen sulfide and panquinone, catalyzing the oxidation of hydrogen sulfide into sulfide [37]. Pyridine nucleotide disulfide reductase is involved in the catalytic oxidation of elemental sulfur [38]. The isodisulfide reductase complex plays a leading role in the oxidation of elemental sulfur [39]. The results indicate that Hg^2+^ improves the partial sulfur catalytic oxidation function of *A. ferrooxidans* ATCC 23270. The expression of cytochrome *c* oxidase was significantly increased under the influence of Hg^2+^, and this enzyme is involved in the reduction of Hg^2+^, indicating that the mercury reduction function of *A. ferrooxidans* ATCC 23270 in the presence of Hg^2+^ may depend on this pathway.

The expression of the Hg^2+^ transporter and Hg^2+^ reductase related to the cellular stress detoxication mechanism was upregulated in the Fe^2+^ oxidation group (Figure 9b). The results indicate that the presence of Hg^2+^ inhibited the overall growth and metabolism function of *A. ferrooxidans* ATCC 23270, but the expression of mercury reduction-related functional genes was active. In the S^0^ oxidation system (Figure 9b), the effects of Hg^2+^ on the expression levels of various functional genes of *A. ferrooxidans* ATCC 23270 were mainly regulated, and only the expression levels of cell-related functional genes were downregulated. Studies have shown that *A. ferrooxidans* ATCC 23270 can increase the activities of superoxide dismutase, glutathione reductase, and thioredoxin reductase due to stress behavior when leaching sulfide ore containing heavy metal ions [40]. Therefore, the upregulated expression of glutathione-related functional genes indicates that *A. ferrooxidans* ATCC 23270 has an active sulfur oxidation function in the system solely metabolized with S^0^ as the substrate, and the detoxification mechanism of Hg^2+^ is jointly produced by glutathione-related mercapto coupling and the reduction of Hg^2+^ reductase.

Compared with the control group with Hg^2+^ (SF0b), Hg^2+^ stress inhibited the synthesis of logarithmic preglycosyltransferase I and a variety of unknown proteins and promoted the expressions of cytochrome *c* (Cyc2), cytochrome *c_552_* (Cyc1), thioredoxin-dependent adenylate sulfate reductase (CysH), and other genes (Figure 9c, Table S2). Cyc2 and Cyc1 participate in the electronic chain transfer pathway for the *A. ferrooxidans* ATCC 23270 ferrous oxidation group, and studies have shown that the two cytochrome *c* genes are related to anaerobic iron reduction coupled with sulfur oxidation [41]. Cytochrome *c* oxidase downstream of the electron transport chain can participate in the reduction of Hg^2+^ [18]. CysH is involved in the sulfur reduction process in sulfur metabolism. The above results indicate that, under the influence of Hg^2+^, *A. ferrooxidans* ATCC 23270 has a low expression of glycosyltransferase, indicating that Hg^2+^ has a negative effect on its biomolecular function. The upregulated expression of cytochrome *c* oxidase also promoted the expressions of functional genes of iron and sulfur metabolism, which had positive effects on the detoxification mechanism of Hg^2+^, the activity of iron and sulfur metabolism, and the motor function of bacteria.

When the bacteria in the experimental group grew to the middle and late logarithmic stages, the total amount of differential genes was very small compared with that in the control group, indicating that, with the growth of bacteria and the progress of mercury conversion, the stress effect of external Hg^2+^ on bacteria was reduced such that they could return to normal growth and metabolism levels (Figure 9d). The expression levels of a few genes related to the localization function of biological processes, cell surface protein complexes, and molecular transport activities were downregulated, suggesting that the metabolic function of bacterial cells was still affected to some extent after most of the Hg^2+^ stress was relieved.

*A. ferrooxidans* ATCC 23270 gene expressions in the aerobic stage and anaerobic stage were compared (Figure 9e, Tables S3 and S4). *A. ferrooxidans* ATCC 23270 was greatly influenced by Hg^2+^ in the aerobic stage, which mainly manifested in the enhanced expression of functional genes related to mercury conversion. The expression of functional genes related to physiological processes such as transcription and translation was reduced. After entering the anaerobic stage, due to the change in oxygen concentration, the electron transfer pathway of the *A. ferrooxidans* ATCC 23270 metabolic process changed greatly, resulting in a great difference between the expressions of each gene and the samples in the aerobic stage. However, there was little difference in gene expression between different cases in the anaerobic stage, indicating that the influence of Hg^2+^ was light in this stage, which is consistent with previous experimental results. In addition, the expression of Hg^2+^ reductase gradually increased and then decreased over time, indicating that the stress effect of Hg^2+^ on *A. ferrooxidans* ATCC 23270 was enhanced in the late aerobic stage and then weakened in the anaerobic stage.

*A. ferrooxidans* ATCC 23270 showed an increase in differentially expressed genes in the middle and late stages of the aerobic stage, and the overall level was downregulated, while the expressions of *merC* and *merA* were upregulated (Figure 9f). The results indicate that the middle and late stages of the aerobic stage are greatly influenced by Hg^2+^. *A. ferrooxidans* ATCC 23270 cells greatly improved the fixation, transfer and reduction function of Hg^2+^. The overall differential gene expression of *A. ferrooxidans* ATCC 23270 affected by Hg^2+^ showed a downward trend compared with the control group (Figure 9f). However, in the late anaerobic stage, the total amount of differentially expressed genes was significantly reduced, indicating that *A. ferrooxidans* ATCC 23270 essentially completed the process of detoxification and developed tolerance to Hg^2+^ under anaerobic conditions. However, because there was still a small amount of Hg^2+^ in the solution, the expressions of *merC* and *merA* showed upregulated trends compared with the control group.

### 3.7. Correlation between the Iron/Sulfur Redox Mediated by Acidithiobacillus ferrooxidans ATCC 23270 and Mercury Transformation Therein

**Figure 10:**
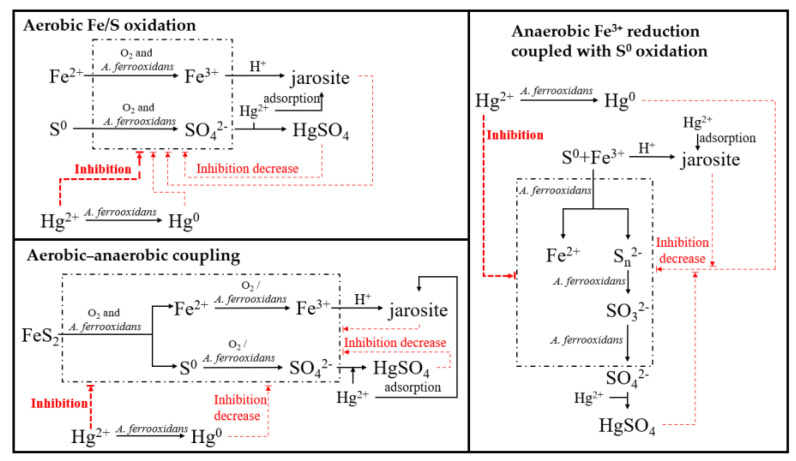
Correlation between the iron/sulfur redox mediated by *Acidithiobacillus ferrooxidans* ATCC 23270 and the mercury transformation therein. The black dashed boxes indicate the iron/sulfur redox process of *A. ferrooxidans* ATCC 23270, the black solid lines indicate the formation of the intermediate and/or products, and red dashed lines indicate the inhibition of bacterial activity by mercury.

The present study shows that the iron/sulfur redox of *Acidithiobacillus ferrooxidans* ATCC was obviously affected by the addition of Hg^2+^, and mercury transformation was thus closely related to the energy substrates and the growth conditions; the proposed correlation mechanism is shown in Figure 10.

It was found that, for all the groups of *A. ferrooxidans* ATCC 23270 grown on different energy substrates under different aerobic and anaerobic conditions, Hg^2+^ significantly inhibited cell growth and reproduction. For the groups with S^0^, Fe^2+^, and Fe^3+^/S^0^ as energy substrates, the inhibition effects were gradually reduced as the culture time increased, and they were eliminated at the late stage of the experiment. Compared with the simple aerobic/anaerobic environment with S^0^, Fe^2+^, and Fe^3+^/S^0^ as the energy substrates, the aerobic–anaerobic coupling environment simulating the AMD environment was more complex. However, due to the adsorption effect of pyrite on Hg^2+^, the aerobic–anaerobic coupling group had little influence on the growth and metabolism and other physiological processes of *A. ferrooxidans* ATCC 23270 at the beginning. Later, with the utilization of the substrate pyrite by *A. ferrooxidans* ATCC 23270, the Hg^2+^ adsorbed on the surface of pyrite was gradually released, gradually inhibiting the growth and reproduction of bacteria. At the same time, the contents of extracellular polymers increased and changed. The bonding mode and distribution on the bacterial surface also changed. At the same time, *A. ferrooxidans* ATCC 23270 enhanced the functions of Hg^2+^ stress, mercury transformation, and intercellular signal transduction.

It was also found that Hg^2+^ significantly changed the composition of polymer on the surface of *A. ferrooxidans* ATCC 23270 and the distribution of C, N, Fe, S, and other elements inside the cells. Particularly in the group containing substrate S, *A. ferrooxidans* ATCC 23270 was more likely to enrich Hg outside and inside the cell walls due to the affinity of S to Hg. Furthermore, *A. ferrooxidans* ATCC 23270 can change the form of Hg, transfer it in the solution and solid phases through iron sulfur redox coupled with mercury conversion, and participate in the mercury cycle together with other mercury-tolerant bacteria. In mercury conversion, the bacteria attached Hg elements to the surface of the substrate sulfur residue or the iron vitriol dregs in the form of Hg^0^, HgS, and HgSO_4_ in all groups to reduce the concentration of Hg^2+^ in solution. Hg^2+^ combined with the SO_4_^2−^ generated during the metabolism of iron and sulfur to form HgSO_4_. In addition, secondary products, such as jarosite, generated by the oxidation of Fe^2+^ can also adsorb Hg^2+^ in the solution. At the same time, Hg^2+^ can also be transported into the cell under the action of *A. ferrooxidans* ATCC 23270 and converted into Hg^0^ by the mercury reduction of *A. ferrooxidans* ATCC 23270. Hg^0^ will combine with S^0^ to produce HgS. However, *A. ferrooxidans* ATCC 23270 underwent slightly different mercury reduction pathways in the three groups. In the aerobic system, the sulfur oxidation group mainly adsorbed and transformed the Hg^2+^ in solution through the glutathione-associated sulfhydryl coupling pathway, *mer* operon-related mercury reduction, and mercury reduction associated with cytochrome *c* oxidase. The mercury reduction pathways in the other three groups were related to *mer* operons.

Notably, the responses of the bacteria to the three different conditions were different. Under single aerobic or anaerobic conditions, the expressions of functional genes related to mercury transformation and the iron/sulfur redox of *A. ferrooxidans* ATCC 23270 increased. Under the aerobic–anaerobic coupling conditions, the strong adsorption of pyrite on Hg^2+^ enhanced the continuous stress of the Hg element, which significantly inhibited the expression of iron and sulfur metabolism function genes of *A. ferrooxidans* ATCC 23270 in the middle and late aerobic stages. However, this gradually recovered during the anaerobic phase.

## 4. Conclusions

This work studied the mercury transformation behavior coupled with iron/sulfur redox mediated by *A. ferrooxidans* ATCC 23270 under aerobic, anaerobic, and coupled aerobic–anaerobic conditions. The presence of Hg^2+^ significantly inhibited microbial growth by decreasing the iron/sulfur redox activities of *A. ferrooxidans* ATCC 23270, but the cells grown under aerobic–anaerobic coupling conditions could more quickly release this inhibition than other groups due to the strong adsorption of pyrite on Hg^2+^. The addition of Hg^2+^ caused a significant change in the composition of bacterial surface compounds and elements such as C, N, S, and Fe, and thus affected the speciation transformation of Hg and S, where Hg was mainly present in the form of Hg^0^, HgS, and HgSO_4_ in the solid substrate residues for all groups. The presence of Hg^2+^ seriously inhibited the expression of functional genes relevant to the iron and sulfur metabolism of *A. ferrooxidans* ATCC 23270, and the expression of mercury-resistant genes was higher in earlier stages of growth than in the later stages of growth for the groups with aerobic iron and sulfur oxidation and the anaerobic Fe^3+^ reduction coupled with S^0^ oxidation. For the aerobic–anaerobic coupling group, when comparing with gene expression at the aerobic stage, we can see that the expression level of mercury reduction genes increased, and the stress effect was weakened after *A. ferrooxidans* ATCC 23270 entered the anaerobic stage.

## Data Availability

Not applicable.

## Supplementary Materials

The following supporting information can be downloaded at: https://www.mdpi.com/article/10.3390/microorganisms11041028/s1, Figure S1: XRD patterns of original sulfur and pyrite; Figure S2: [Total Fe] and ORP of solution for *A. ferrooxidans* ATCC 23270 with different concentrations of Hg^2+^; Figure S3: TEM-EDS images of bacterial cells for *A. ferrooxidans* ATCC 23270 without Hg^2+^; Figure S4: XPS images of bacterial cells for *A. ferrooxidans* ATCC 23270 in the presence of Hg^2+^; Table S1: Genes related to mercury transformation in iron/sulfur metabolism during the oxidation of S^0^/Fe^2+^ by *A. ferrooxidans* ATCC 23270 in the presence of Hg^2+^; Table S2: Genes related to mercury transformation in iron/sulfur metabolism during the anaerobic Fe^3+^ reduction coupled with S^0^ oxidation of *A. ferrooxidans* ATCC 23270 in the presence of Hg^2+^; Table S3: Genes related to mercury transformation in iron/sulfur metabolism during A. ferrooxidans ATCC 23270–pyrite interaction under aerobic conditions in the presence of Hg^2+^; Table S4: Genes related to mercury transformation in iron/sulfur metabolism during *A. ferrooxidans* ATCC 23270–pyrite interaction under anaerobic conditions in the presence of Hg^2+^. References [42,43,44,45] are cited in the Supplementary Materials.
