# Supplementary material for: Fe/S Redox-Coupled Mercury Transformation Mediated by Acidithiobacillus ferrooxidans ATCC 23270 under Aerobic and/or Anaerobic Conditions
Source: Microorganisms. 2023 Apr 14;11(4):1028. doi: 10.3390/microorganisms11041028 (PMC10141921; doi:10.3390/microorganisms11041028)
Supplement: Supplementary file 1 [file microorganisms-11-01028-s001.zip › microorganisms-2292908-supplementary.pdf]

microorganisms

MDPI

Article

Fe/S Redox-Coupled Mercury Transformation Mediated by *Acidithiobacillus ferrooxidans* ATCC 23270 under Aerobic and/or Anaerobic Conditions

Yue Liu [1,†], Chenyun Gu [1,†], Hongchang Liu [1,2,*], Yuhang Zhou [1], Zhenyuan Nie [1,2], Yirong Wang [1] and Lu Chen [1], Jinlan Xia [1,2,*] and Wensheng Shu [3]

[1] School of Minerals Processing and Bioengineering, Central South University, Changsha 410083, China
[2] Key Lab of Biometallurgy of Ministry of Education of China, Central South University, Changsha 410083, China
[3] School of Life Science, South China Normal University, Guangzhou 510631, China
* Correspondence: hchliu2050@csu.edu.cn; jlxia@csu.edu.cn
† These authors have contributed equally to this work

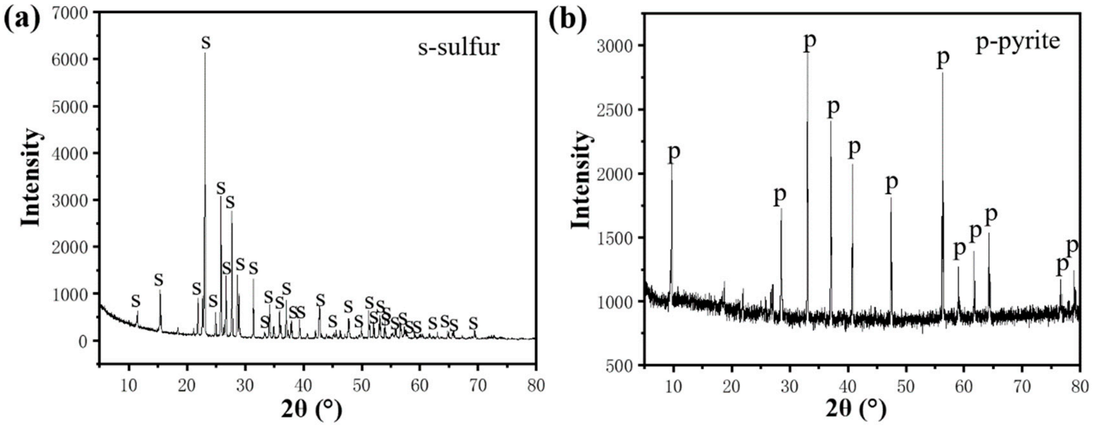

Figure S1. XRD patterns of original sulfur (a) and pyrite (b).

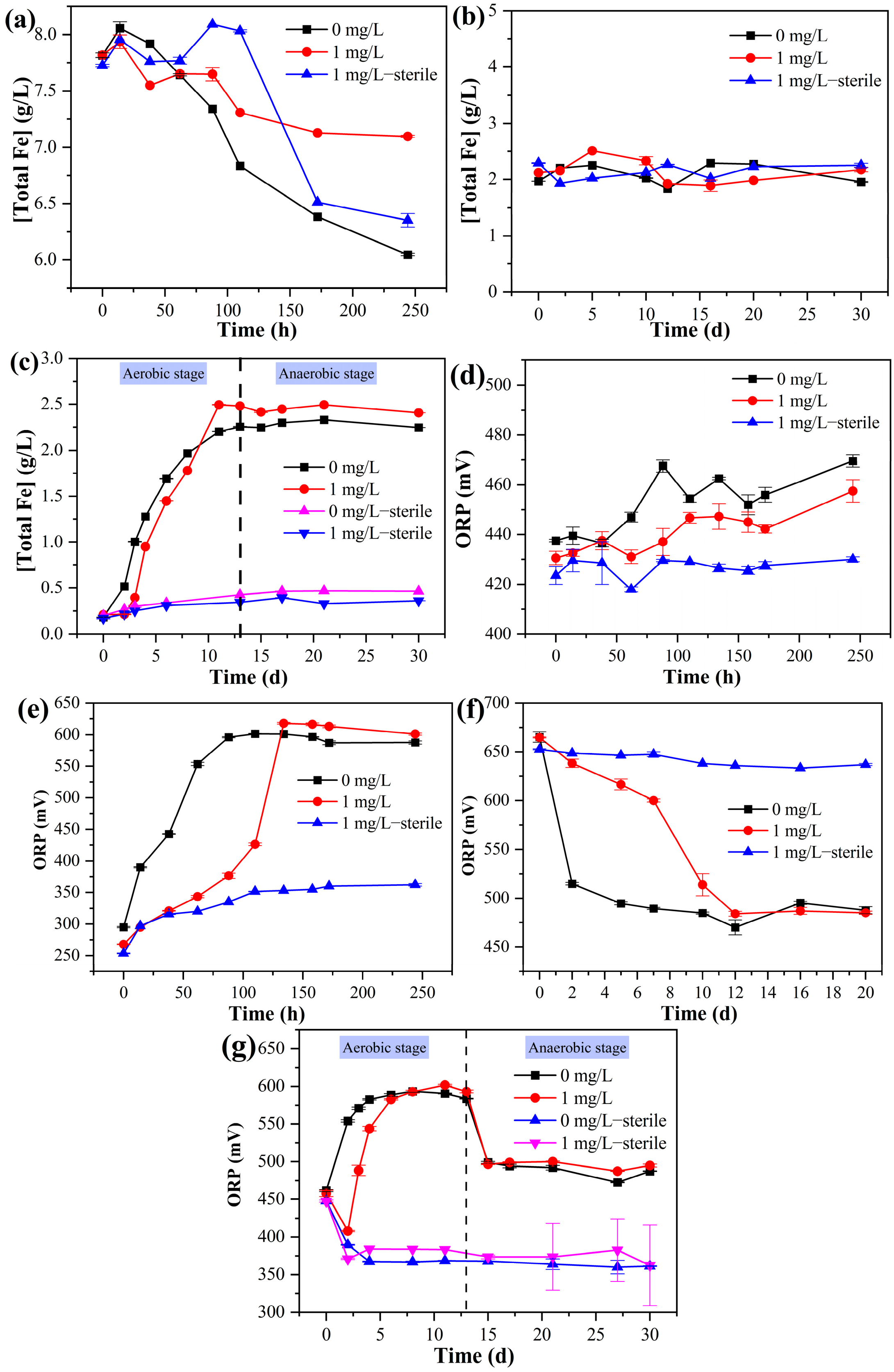

Figure S2. [Total Fe] and ORP of solution by *A. ferrooxidans* ATCC 23270 with different concentrations of Hg^{2+}. (a) [Total Fe] in Fe^{2+} oxidation; (b) [Total Fe] in anaerobic Fe^{3+} reduction coupled S^0 oxidation; (c) [Total Fe] in aerobic–anaerobic coupling; (d) ORP of Fe^{2+} oxidation; (e) ORP of S^0 oxidation; (f) ORP of anaerobic Fe^{3+} reduction coupled S^0 oxidation; (g) ORP of aerobic–anaerobic coupling.

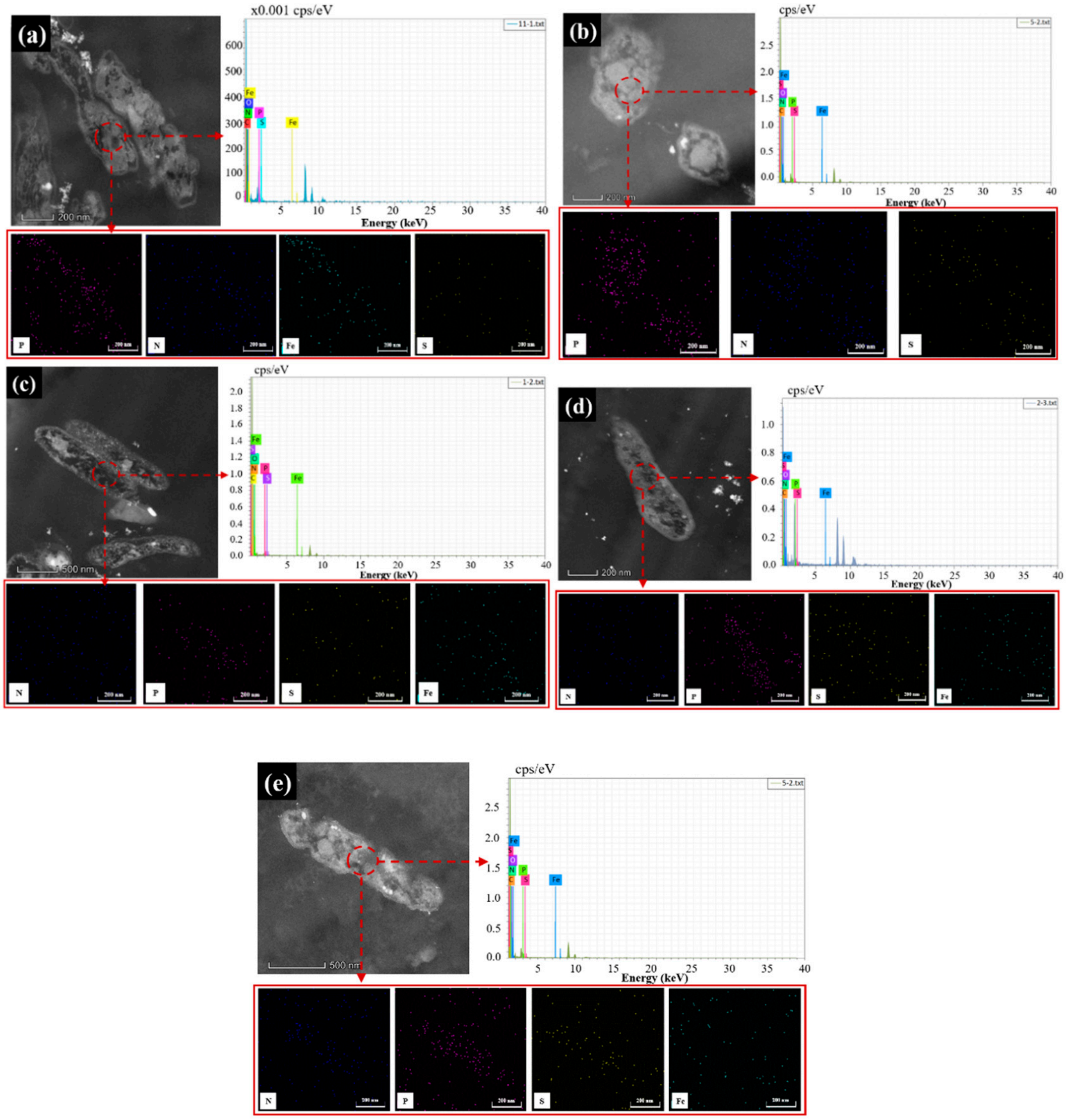

Figure S3. TEM-EDS images of bacterial cells by *A. ferrooxidans* ATCC 23270 without Hg^{2+}. (a) Fe^{2+} oxidation system; (b) S^0 oxidation; c: anaerobic Fe^{3+} reduction coupled S^0 oxidation; (d) the aerobic stage of aerobic–anaerobic coupling; (e) the anaerobic stage of aerobic–anaerobic coupling.

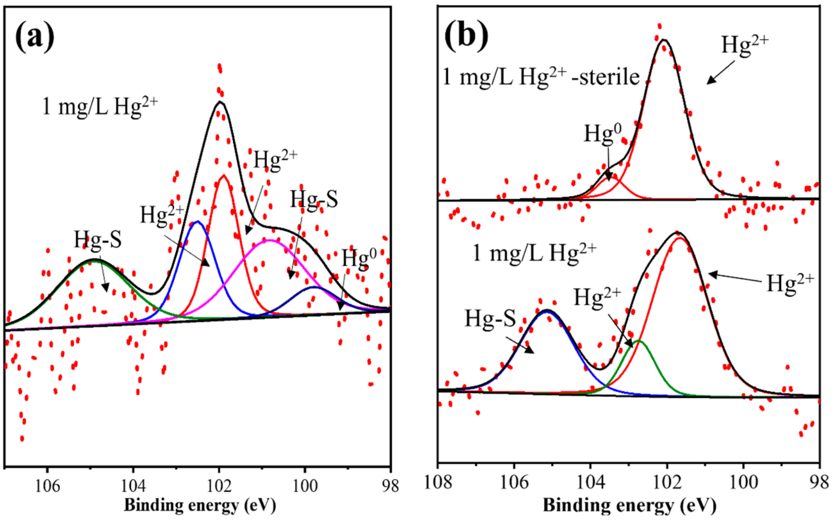

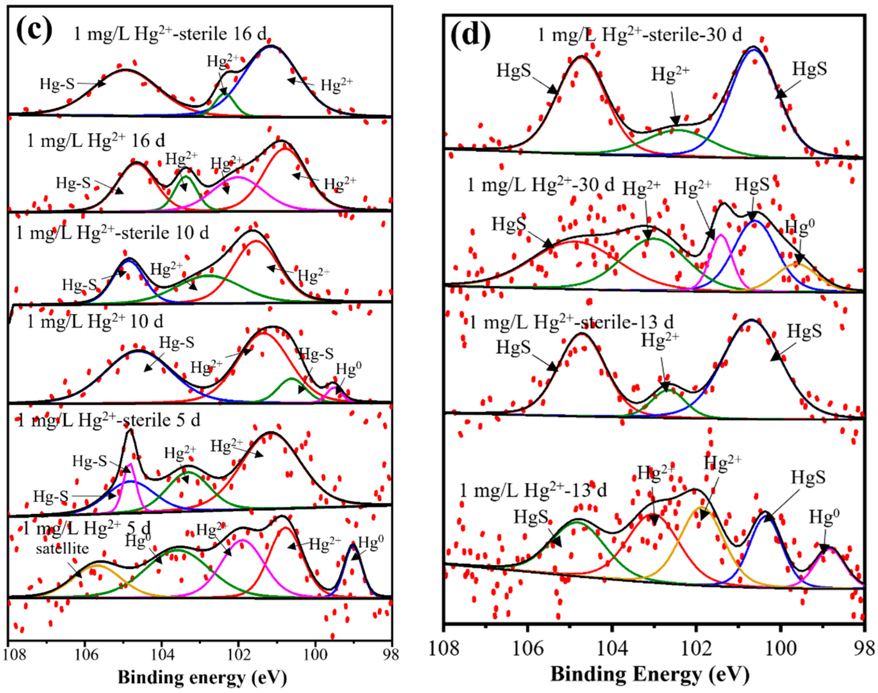

Figure S4. XPS images of bacterial cells by *A. ferrooxidans* ATCC 23270 in the presence of Hg^{2+}. (a) Fe^{2+} oxidation; (b) S^0 oxidation; (c) anaerobic Fe^{3+} reduction coupled S^0 oxidation; (d) the aerobic stage of aerobic–anaerobic coupling; (e) the anaerobic stage of aerobic–anaerobic coupling.

Note: The XPS spectral signal-to-noise ratio of Hg4f is low because there is very little Hg element on the substrate residue surface for the Fe^{2+} oxidation group (Figure S4(a)). It can be concluded that the Hg element on the surface of the substrate residue may mainly exist in the form of Hg^{2+}, Hg^0 and HgS, and the fitting peak area of HgS is the highest, confirming the existence of the Hg-S bond. The substrate residue for the S^0 oxidation group showed that the Hg element on the substrate residue surface of the bacterial group and the sterile group with Hg^{2+} was mainly composed of Hg^{2+} (101.8 eV, 102.6 eV), Hg^0 (99.8 eV, 104.0 eV) and HgS (104.8 eV) (Figure. S4(b)) [42–45]. This indicates that the bacteria are continually reducing Hg^{2+} to Hg^0. Some low valence sulfur on the surface of S^0 combined with Hg^{2+} to generate HgS precipitation. The form transformation of S, Fe and Hg is closely related to the Fe/S oxidation of *A. ferrooxidans* ATCC 23270. *A. ferrooxidans* ATCC 23270 oxidizes S^0/Fe^{2+} under related aerobic conditions, resulting in a change in S/Fe morphology, producing SO_4^{2-}/Fe^{3+}. Most of the SO_4^{2-} exists in the free form in solution, and combines with Hg^{2+} to form $HgSO_4$, which reduces the concentration of Hg^{2+} in solution. Fe^{3+} combines with the ions in the solution and settles into jarosite to absorb the remaining Hg^{2+} in the solution that has not been transformed by bacteria.

For the groups with Hg^{2+}, the Hg element on the surface of the substrate residue also mainly exists in the form of HgS, Hg^{2+} and Hg^0 (Figure. S4(c)) [42–45]. Hg in the sterile control group mainly existed in the form of HgS and Hg^{2+}, while the fitting peak of Hg^0 appeared successively for the bio group at day 5 and day 10, and almost disappeared completely at day 16. It is confirmed that *A. ferrooxidans* ATCC 23270 can transform mercury by reducing Hg^{2+} to Hg^0, and the product Hg^0 is combined with S^0 in the substrate residue to generate HgS, while HgS in the sterile group mainly comes from the combination of Hg^{2+} in solution and the low valence sulfur on the substrate S^0 residue surface.

The aerobic–anaerobic group is the same as the previous systems, in which a small amount of Hg exists on the surface of the substrate residue in the form of HgS, Hg^{2+} and Hg^0 (Figure. S4(d)) [42–45]. There were more fitting peaks of Hg on the substrate residue surface for the bio group, and the signal-to-noise ratio of spectral lines was low, while there were fewer fitting peaks and high peak intensity of Hg in the sterile control experiments. This indicates that Hg

is relatively small on the substrate residue surface for the bio group, and the form of Hg is abundant under the action of bacterial transformation, while the form of Hg is relatively simple in the sterile control group, and Hg^{2+} is mainly in the form of HgS adsorbed on the surface of pyrite. In addition, with the passage of time, the half-peak width and proportion of HgS fitting peak on the substrate residue surface for the bio group are increasing, indicating that the final Hg element tends to combine with S on the surface of pyrite under the coupling of the bacterial mercury conversion process and iron-sulfur oxidation.

Table S1. Genes related to mercury transformation in iron/sulfur metabolism during the oxidation of S^0/Fe^{2+} by *A. ferrooxidans* ATCC 23270 in the presence of Hg^{2+}.

Description	Gene_ID	S1b vs S0 [1]	F1b vs F0 [2]
mercuric resistance protein *MerC*	*AFE_2480*	up-regulation	up-regulation
mercuric reductase	*AFE_2481*	up-regulation	up-regulation
glutathione S-transferase	*AFE_2594*	up-regulation	down-regulation
rusticyanin	*AFE_3146*	up-regulation	up-regulation
cytochrome *c* oxidase, aa_3-type(cox)	*AFE_3147*<br>*AFE_3148*<br>*AFE_3149*<br>*AFE_3150*	up-regulation	-
cytochrome c_{552}	*AFE_3152*	up-regulation	up-regulation
cytochrome *c*	*AFE_1428*<br>*AFE_3153*	up-regulation	up-regulation
cytochrome c_4	*AFE_2727*<br>*AFE_3107*	up-regulation	up-regulation
cytochrome *c* reductase (pet)	*AFE_2729*<br>*AFE_2730*<br>*AFE_2731*	up-regulation	-
heterodisulfide reductase	*AFE_2554*<br>*AFE_2586*	up-regulation	up-regulation

[1] S1b vs S0: S^0 oxidation system 1 mg/L [Hg^{2+}] vs 0 mg/L [Hg^{2+}]. [2] F1b vs F0: Fe^{2+} oxidation system 1 mg/L [Hg^{2+}] vs 0 mg/L [Hg^{2+}].

Table S2. Genes related to mercury transformation in iron/sulfur metabolism during the anaerobic Fe^{3+} reduction coupled S^0 oxidation of *A. ferrooxidans* ATCC 23270 in the presence of Hg^{2+}.

Description	Gene_ID	SF1a vs SF0b [1]	SF1b vs SF0b [2]
mercuric resistance protein *MerC*	*AFE_2480*	up-regulation	up-regulation
mercuric reductase	*AFE_2481*	up-regulation	up-regulation
rusticyanin	*AFE_3146*	up-regulation	up-regulation
cytochrome *c* oxidase, aa_3-type(cox)	*AFE_3148*<br>*AFE_3149*<br>*AFE_3150*<br>*AFE_3147*	up-regulation	down-regulation
cytochrome c_{552}	*AFE_3152*	up-regulation	down-regulation
cytochrome *c*	*AFE_1428*<br>*AFE_3153*	up-regulation	up-regulation

cytochrome c_4	*AFE_2727*<br>*AFE_3107*	up-regulation	up-regulation
cytochrome c reductase (pet)	*AFE_2729*<br>*AFE_2730*<br>*AFE_2731*	up-regulation	up-regulation

[1] SF1a vs SF0b: 1 mg/L [Hg^{2+}] at prometaphase vs 0 mg/L [Hg^{2+}]. [2] b: 1 mg/L [Hg^{2+}] at last stage vs 0 mg/L [Hg^{2+}].

Table S3. Genes related to mercury transformation in iron/sulfur metabolism during *A. ferrooxidans* ATCC 23270-pyrite interaction under aerobic conditions in the presence of Hg^{2+}.

Description	Gene_ID	Mo1a vs Mo0a [1]	Mo1b vs Mo0a [2]
mercuric resistance protein MerC	*AFE_2480*	up-regulation	up-regulation
mercuric reductase	*AFE_2481*	up-regulation	up-regulation
rusticyanin	*AFE_3146*	down-regulation	down-regulation
cytochrome *c* oxidase, *aa*$_3$-type(cox)	*AFE_3147*<br>*AFE_3148*<br>*AFE_3149*<br>*AFE_3150*	down-regulation	down-regulation
cytochrome *c*	*AFE_3107*<br>*AFE_3152*<br>*AFE_3153*	down-regulation	down-regulation
cytochrome c_4	*AFE_2727*	up-regulation	up-regulation
cytochrome *c* reductase (pet)	*AFE_2729*<br>*AFE_2730*<br>*AFE_2731*	up-regulation	up-regulation
heterodisulfide reductase	*AFE_2551*<br>*AFE_2553*<br>*AFE_2554*<br>*AFE_2586*	up-regulation	up-regulation
sulfate adenylyltransferase	*AFE_0539*	down-regulation	down-regulation

[1] Mo1a vs Mo0a: 1 mg/L [Hg^{2+}] at prometaphase of aerobic phase vs 0 mg/L [Hg^{2+}] at aerobic phase.

[2] Mo1b vs Mo0a: 1 mg/L [Hg^{2+}] at last stage of aerobic phase vs 0 mg/L [Hg^{2+}] at aerobic phase.

Table S4. Genes related to mercury transformation in iron/sulfur metabolism during *A. ferrooxidans* ATCC 23270-pyrite interaction under anaerobic conditions in the presence of Hg^{2+}.

Description	Gene_ID	Ma1a vs Ma0a [1]	Ma1b vs Ma0a [2]
mercuric resistance protein MerC	*AFE_2480*	up-regulation	up-regulation
mercuric reductase	*AFE_2481*	up-regulation	up-regulation
rusticyanin	*AFE_3146*	down-regulation	down-regulation
cytochrome *c* oxidase, *aa*$_3$-type(cox)	*AFE_3147*<br>*AFE_3148*<br>*AFE_3149*<br>*AFE_3150*	down-regulation	down-regulation

cytochrome *c*	*AFE_3152*<br>*AFE_3153*	down-regulation	down-regulation
cytochrome *c4*	*AFE_3107*<br>*AFE_2727*	up-regulation	up-regulation
cytochrome c reductase (pet)	*AFE_2729*<br>*AFE_2730*<br>*AFE_2731*	down-regulation	down-regulation

[1] Ma1a vs Ma0a: 1 mg/L [Hg^{2+}] at prometaphase of anaerobic phase vs 0 mg/L [Hg^{2+}] at anaerobic phase.

[2] Ma1b vs Ma0a: 1 mg/L [Hg^{2+}] at last stage of anaerobic phase vs 0 mg/L [Hg^{2+}] at anaerobic phase.

References

42. Huang, Z.; Wei, Z.; Xiao, X.; Tang, M.; Li, B.; Zhang, X. Nitrification/Denitrification Shaped the Mercury-Oxidizing Microbial Community for Simultaneous Hg0 and NO Removal. *Bioresour. Technol.* **2019**, *274*, 18–24. https://doi.org/10.1016/j.biortech.2018.11.069.
43. Huang, Z.; Wei, Z.; Xiao, X.; Tang, M.; Li, B.; Ming, S.; Cheng, X. Bio-Oxidation of Elemental Mercury into Mercury Sulfide and Humic Acid-Bound Mercury by Sulfate Reduction for Hg0 Removal in Flue Gas. *Environ. Sci. Technol.* **2019**, *53*, 12923–12934. https://doi.org/10.1021/acs.est.9b04029.
44. Wang, M.; Li, Y.; Zhao, D.; Zhuang, L.; Yang, G.; Gong, Y. Immobilization of Mercury by Iron Sulfide Nanoparticles Alters Mercury Speciation and Microbial Methylation in Contaminated Groundwater. *Chem. Eng. J.* **2020**, *381*, 122664. https://doi.org/10.1016/j.cej.2019.122664.
45. Hu, L.; Liu, B.; Li, S.; Zhong, H.; He, Z. Study on the Oxidative Stress and Transcriptional Level in Cr(VI) and Hg(II) Reducing Strain Acinetobacter Indicus Yy-1 Isolated from Chromium-Contaminated Soil. *Chemosphere* **2021**, *269*, 128741. https://doi.org/10.1016/j.chemosphere.2020.128741.
